# N6‐methyladenosine reader YTHDF3‐mediated zinc finger protein 41 inhibits hepatocellular carcinoma progression by transcriptional repression of Snail

**DOI:** 10.1002/mco2.763

**Published:** 2024-10-28

**Authors:** Xinxin Li, Mengzhen Han, He Zhu, Hongwei Zhang, Yonglong Pan, Huifang Liang, Zhibin Liao, Bixiang Zhang, Xiaoping Chen

**Affiliations:** ^1^ Hepatic Surgery Center Tongji Hospital Tongji Medical College Huazhong University of Science and Technology Wuhan Hubei China; ^2^ Hubei Key Laboratory of Hepato‐Pancreato‐Biliary Diseases Wuhan Hubei China

**Keywords:** EMT, hepatocellular carcinoma, m^6^A, transcription regulation, ZFP41

## Abstract

Advanced metastasis of hepatocellular carcinoma (HCC) significantly contributes to high death rates among patients. The efficiency of targeted therapies and chemotherapeutic agents shows individual variability. Therefore, there is no effective treatment for advanced HCC. Zinc finger proteins (ZFPs) are known to be crucial in various tumors, especially on HCC. In our study, we verified that ZFP41 could suppress the progression and metastasis of HCC through in vitro and in vivo experiments. During the past years, N6‐methyladenine (m^6^A) regulation has also been increasingly reported in HCC. To investigate whether ZFP41 could be regulated via m^6^A methylation, our results showed that YTHDF3 bound to the mRNA of ZFP41 and degrade it. Subsequently, to further elucidate the function of ZFP41, we identified Snail, a well‐known oncogenic molecule, through RNA‐seq. As a canonical component in the epithelial‐to‐mesenchymal transition (EMT) pathway, Snail plays a pivotal role and is a critical marker for tumor invasion and metastasis. Our results showed ZFP41 could inhibit Snail and the EMT pathway through its transcriptional repression. In conclusion, our study revealed that ZFP41 is a potential prognostic element for patients with HCC, and targeting ZFP41 might be used for translational clinical applications as a promising therapeutic target.

## Graphical abstract

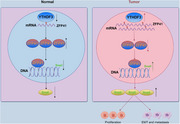

## 1 INTRODUCTION

HCC remains the predominant type of liver cancer, and continues to be the primary cause of tumor‐induced deaths. The primary causes of the elevated mortality rate in advanced HCC are its high likelihood of metastasis and recurrence.^1^ Although current surgical skills have become very advanced, there are still some patients with HCC metastasis or recurrence who are difficult to cure clinically.^2^ Therefore, it becomes particularly important to reveal the pathological progression and molecular mechanism of HCC development.

ZFPs, the most extensive group of transcription factors distinguished by their unique finger‐like DNA binding domains, can play various roles in many pathological processes, especially in cancers.^3^ The functions of ZFPs are also different under diverse environments or stimulations. In tumors, ZFPs can promote or suppress cancer. ZFPs can facilitate or inhibit transcriptional regulation by binding to the DNA of target genes and can also bind to transcription factors at the protein level to assist them in transcriptional regulation.^4^ For example, the overexpression of ZNF143 promotes the progression of the HCC cell cycle by activating CDC6. Specifically, ZNF143 directly activates the transcription of the histone demethylase gene induced by mineral dust, resulting in reduced H3K9me3 accumulation in the CDC6 promoter area.^5^

Epigenetics plays a significant role in gene expression. m^6^A is a reversible alteration of RNA and represents the most abundant modification in mRNA.^6^ Recently, research on m^6^A modification has increased, revealing its significant impact on cell metabolism, tumor immune microenvironment, and alternative splicing.^7^, ^8^, ^9^ YTHDF3, an important “reader,” is crucial in dictating mRNA fate. It has been found that YTHDF3 accelerates aerobic glycolysis and development of HCC by impeding PFKL mRNA degradation via m^6^A modification, and the PFKL protein augments the expression of YTHDF3 protein by inhibiting EFTUD2‐mediated ubiquitination of YTHDF3 protein, thus creating a positive loop between YTHDF3 and PFKL.^10^ m^6^A modification is initiated by a “writer,” and recognized by a “reader,” both of which have a consensus motif “RRACU” (R = G or A). The relationship between ZFPs and m^6^A modification remains unclear. For instance, YTHDF1 promotes the translation of ZFP839, facilitating the binding between ZFP839 and Runx2, thereby enhancing the transcriptional activity of Runx2 and promoting osteogenesis in BMSCs.^11^ Generally, the mechanisms of m^6^A modification in HCC still require more research.^12^

Snail, initially identified as the most significant transcriptional repressor of E‐cadherin, has been reported to be aberrantly expressed in multiple cancer types. This aberrant expression is linked to malignant biological activities, including cell motility, growth, cellular senescence, and programmed cell death.^13^

Our study has discovered that ZFP41 is a downstream target of YTHDF3, which downregulates ZFP41 mRNA level in an m^6^A‐dependent manner. ZFP41 inhibits Snail gene expression by attaching to its promoter area, and the degradation of ZFP41 mRNA by YTHDF3 removes the transcriptional inhibition of ZFP41 to Snail, thus promoting the development of EMT in HCC. Taken together, our research indicates that ZFP41 can serve as a potential biomarker for prognostic diagnosis and a possible therapeutic target in HCC.

## 2 RESULTS

### 2.1 Downregulated ZFP41 is correlated with poor survival in HCC patients

**FIGURE 1:**
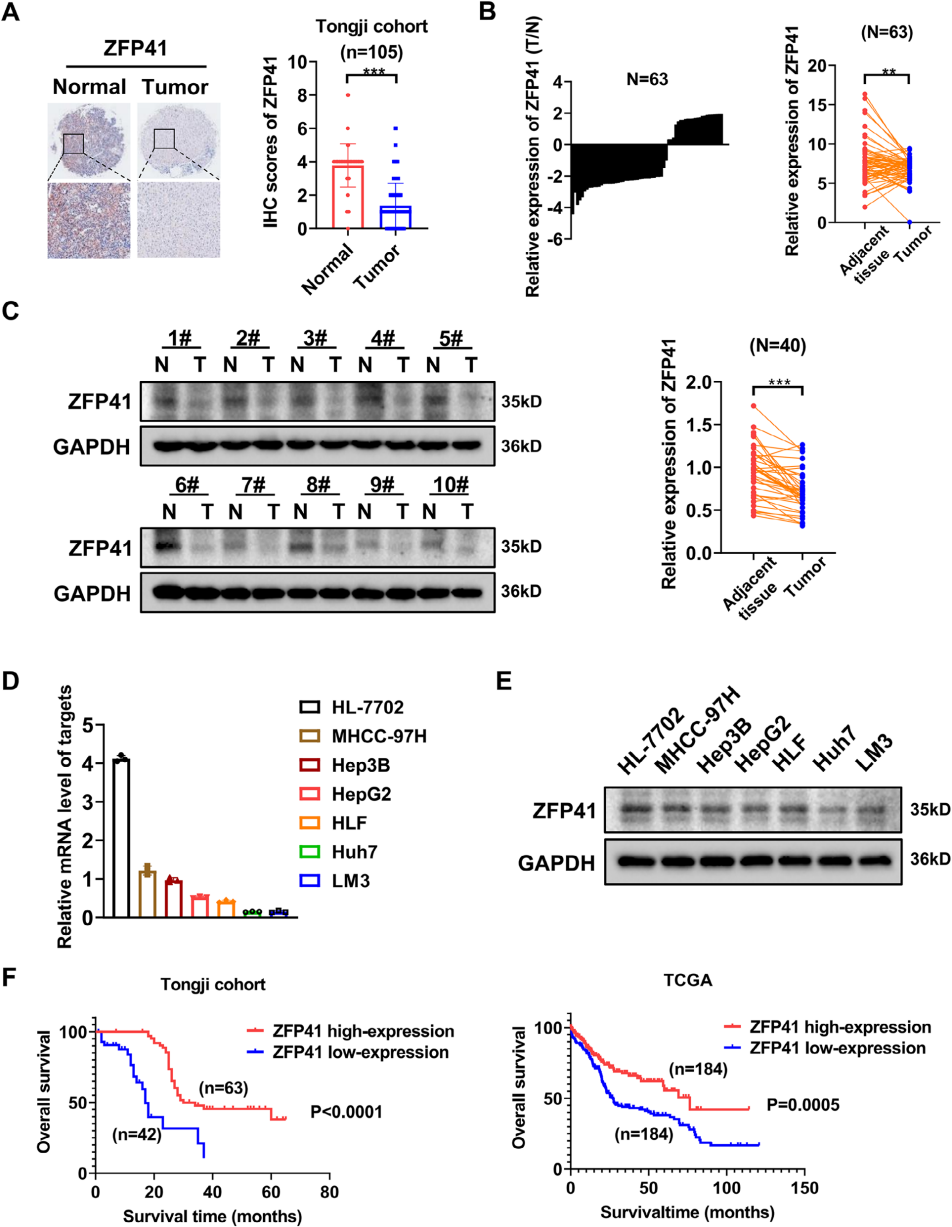
Downregulated ZFP41 is correlated with poor survival in HCC patients. (A) The IHC staining results of tissue microarray from Tongji hospital HCC patients. (B) qPCR results showed that ZFP41 was highly expressed in normal tissues rather than tumor tissues in 63 pairs samples from HCC patients. The relevance of 63 pairs of HCC patient samples was calculated. (C) Western blots results showed the ZFP41 protein level in both tumor and normal tissues. The relevance of 40 pairs of HCC patient samples was demonstrated. (D) qPCR results showed that the mRNA level of ZFP41 in normal hepatocyte cell and other HCC cells. (E) Western blots results showed that the protein level of ZFP41 in normal hepatocyte cell and other HCC cells. (F) The overall survival prognosis of patients determined on the basis of ZFP41 expression in Tongji cohort and TCGA database.

ZFPs play an important role in various types of cancers.^14^, ^15^ Nevertheless, the specific function of ZFP41 in HCC remains unknown.^16^ To explore the function of ZFP41 in HCC progression, we analyzed the immunohistochemical (IHC) staining on tissue arrays that included 105 paired HCC specimens and normal tissues (Figure 1A). The results revealed that ZFP41 expression was obviously elevated in normal tissues compared with the paired HCC specimens. The further to test the expression level of ZFP41 in HCC, its mRNA expression in patient samples was checked. qPCR was conducted to evaluate ZFP41 mRNA expression in 63 pairs of tissue samples from patients. The findings revealed that ZFP41 level was notably lower in HCC tissues compared with normal tissues (*p* < 0.01; Figure 1B). Then, we noticed that ZFP41 protein level was reduced in HCC tissues relative to normal tissues (Figures 1C and S1A). Following this, the expression levels of ZFP41 mRNA and protein in both normal liver and HCC cells were measured. Both qPCR and western blot assay results showed that the expression level of ZFP41 was much higher in HL‐7702 cells than HCC cells (Figure 1D,E). These findings indicated that ZFP41 was overexpressed in normal tissues compared with HCC tissues. Based on the IHC scoring, patients were classified into two groups: a high ZFP41 expression group and a low ZFP41 expression group (Table S2). Consistent with the TCGA database, the findings indicated that patients exhibiting low ZFP41 levels had a worse overall survival (Figure 1F). Overall, the results suggested that the ZFP41 expression was downregulated in HCC and positively associated with the survival time of HCC patients (Table S1).

### 2.2 ZFP41 suppresses HCC cells proliferation in vitro and in vivo

**FIGURE 2:**
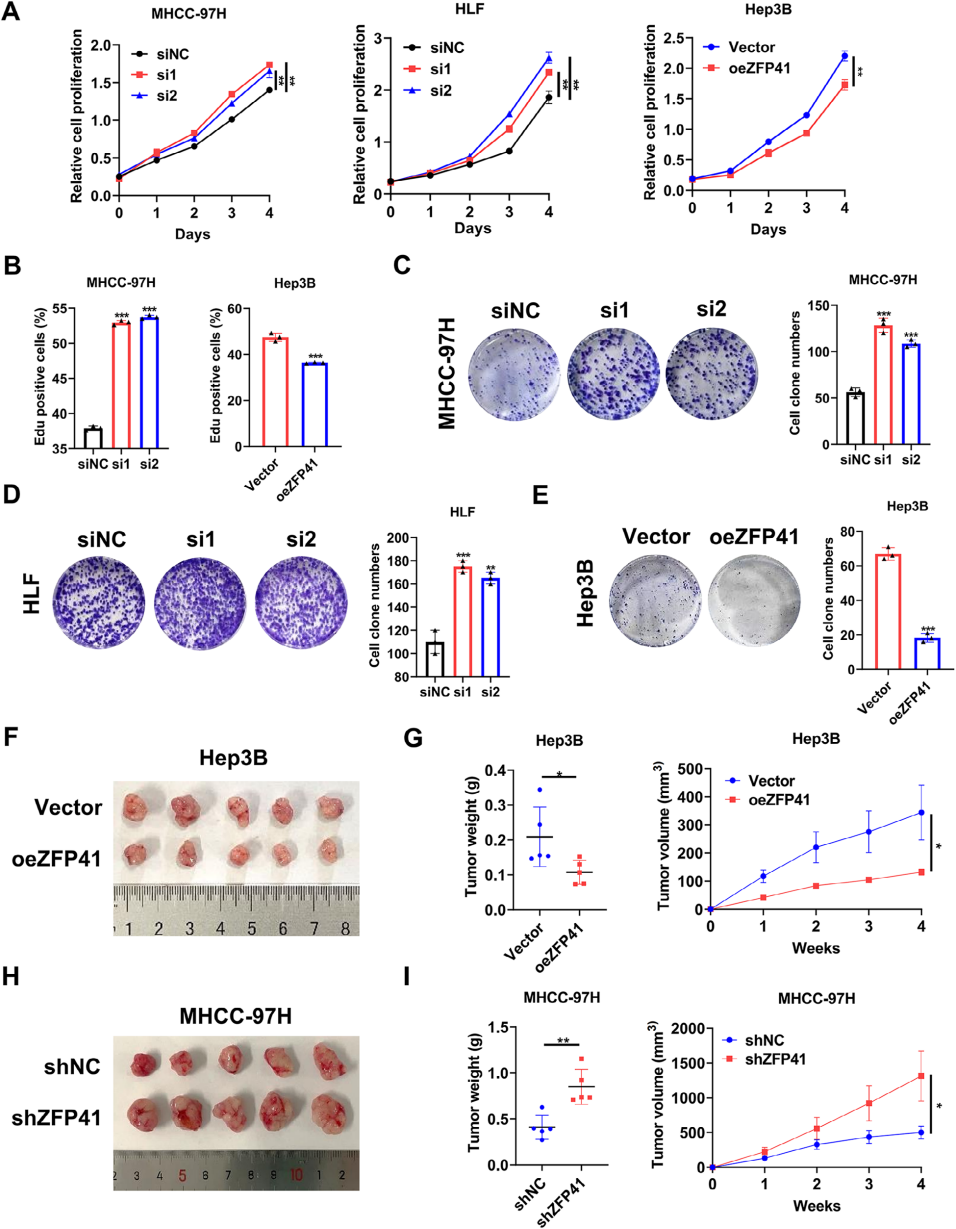
ZFP41 suppresses HCC cells proliferation in vitro and in vivo. (A) CCK‐8 assays in MHCC‐97H, HLF and Hep3B cells with knockdown/overexpression of ZFP41. (B) EdU assays in MHCC‐97H and Hep3B cells with knockdown/overexpression of ZFP41. (C–E) The proliferation effect of MHCC‐97H, HLF, and Hep3B cells with knockdown /overexpression of ZFP41was measured by colony formation assays. (F) The xenograft tumors images of the Vector group and oeZFP41 group in Hep3B cells. (G)The Volumes and Weights of xenograft tumors were measured in Vector group and oeZFP41 group. (H) The xenograft tumors images of xenograft tumors of the shNC group and shZFP41 group in MHCC‐97H cells. (I) Volumes and Weights of xenograft tumors were measured in shNC group and shZFP41 group.

To ascertain the role of ZFP41 in HCC, siRNA transfection was performed to knock down ZFP41 in MHCC‐97H and HLF cells, as well as overexpressed ZFP41 in Hep3B cells. The protein level of ZFP41 was verified following transfection (Figure S2A). In MHCC‐97H and HLF cells, siRNA‐induced knockdown of ZFP41 caused increased cell proliferation and colony numbers. Conversely, the overexpression of ZFP41 in Hep3B cells resulted in reduced cell proliferation and colony numbers (Figure 2A,C–E). Immunofluorescence EdU further demonstrated that knockdown of ZFP41 increased cell growth compared with the control group in MHCC‐97H cells, while upregulation of ZFP41 suppressed Hep3B cells growth (Figures 2B and S2B). We created various xenograft models to assess how ZFP41 influences HCC progression in vivo. First, Hep3B cells overexpressing ZFP41 were subcutaneously injected in nude mice. After 4 weeks, the group with ZFP41 overexpression showed reduced tumor volume and decreased tumor weight relative to the control group (Figure 2F,G). Then, we injected MHCC‐97H cells with ZFP41 knockdown in the same way, the results were opposite (Figure 2H,I). IHC staining demonstrated that overexpression of ZFP41 could decrease the Ki‐67 expression relative to the control group, while reducing ZFP41 resulted in opposite results (Figure S2C,D).

### 2.3 ZFP41 decreases HCC metastasis in vitro and in vivo

**FIGURE 3:**
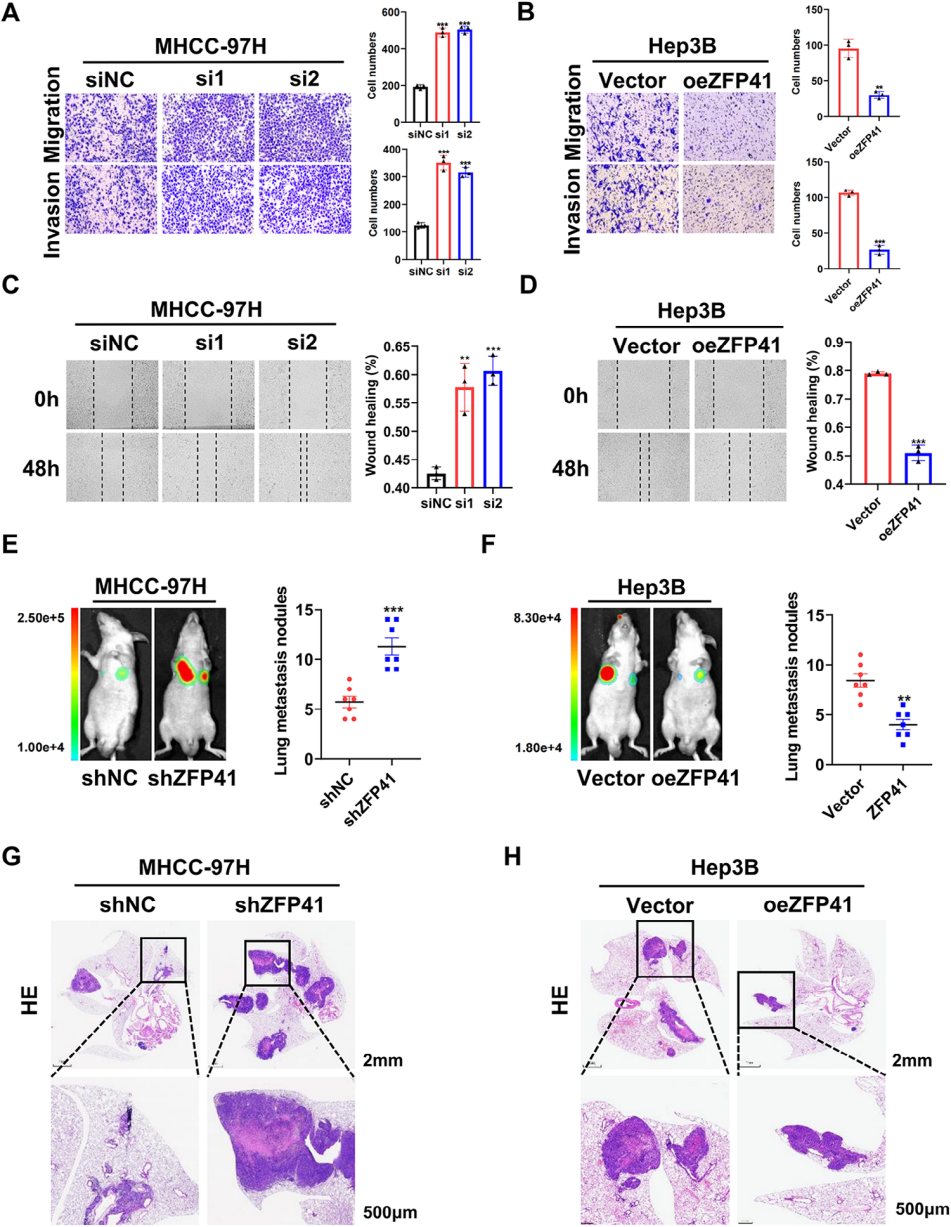
ZFP41 decreases HCC metastasis in vitro and in vivo. (A) The migration and invasion results of MHCC‐97H cells with siRNA‐induced ZFP41 knockdown. (B) The ability of migration and invasion of Hep3B cells with ZFP41 overexpression. (C) The migration rates of MHCC‐97H cells with siRNA‐induced ZFP41 knockdown were showed in wound healing assays. (D) The migration rates of Hep3B cells with ZFP41 overexpression were showed in wound healing assay. (E and F) The lung fluorescence and the number of lung metastatic nodules was calculated in each group. (G and H) HE staining of lung tissue in each group were presented.

To confirm the involvement of ZFP41 in HCC metastasis, experiments for migration and invasion were first conducted in vitro. The result demonstrated that siRNA‐induced knockdown of ZFP41 increased the ability of migration and invasion in MHCC‐97H and HLF cells (Figures 3A and S3A). On the contrary, overexpression of ZFP41 in Hep3B cells resulted in the opposite effects (Figure 3B). Wound healing assays further confirmed these findings (Figures 3C,D and S3B). To evaluate the metastasis effect of ZFP41 in vivo, MHCC‐97H control cells and MHCC‐97H cells with reduced ZFP41 expression were injected via the tail vein in nude mice. After 6 weeks, the mice injected with ZFP41 knockdown cells showed more lesions compared with those injected with control cells (Figure 3E,G). In contrast, overexpression of ZFP41 in Hep3B cells significantly suppressed lung metastasis, as evidenced by a reduced number of nodules compared with the control group (Figure 3F,H). Based on to these results, ZFP41 could repress the development and metastasis of HCC in vitro and in vivo.

### 2.4 YTHDF3‐mediated m^6^A modification of ZFP41 mRNA and its causes in decay of its mRNA stability

**FIGURE 4:**
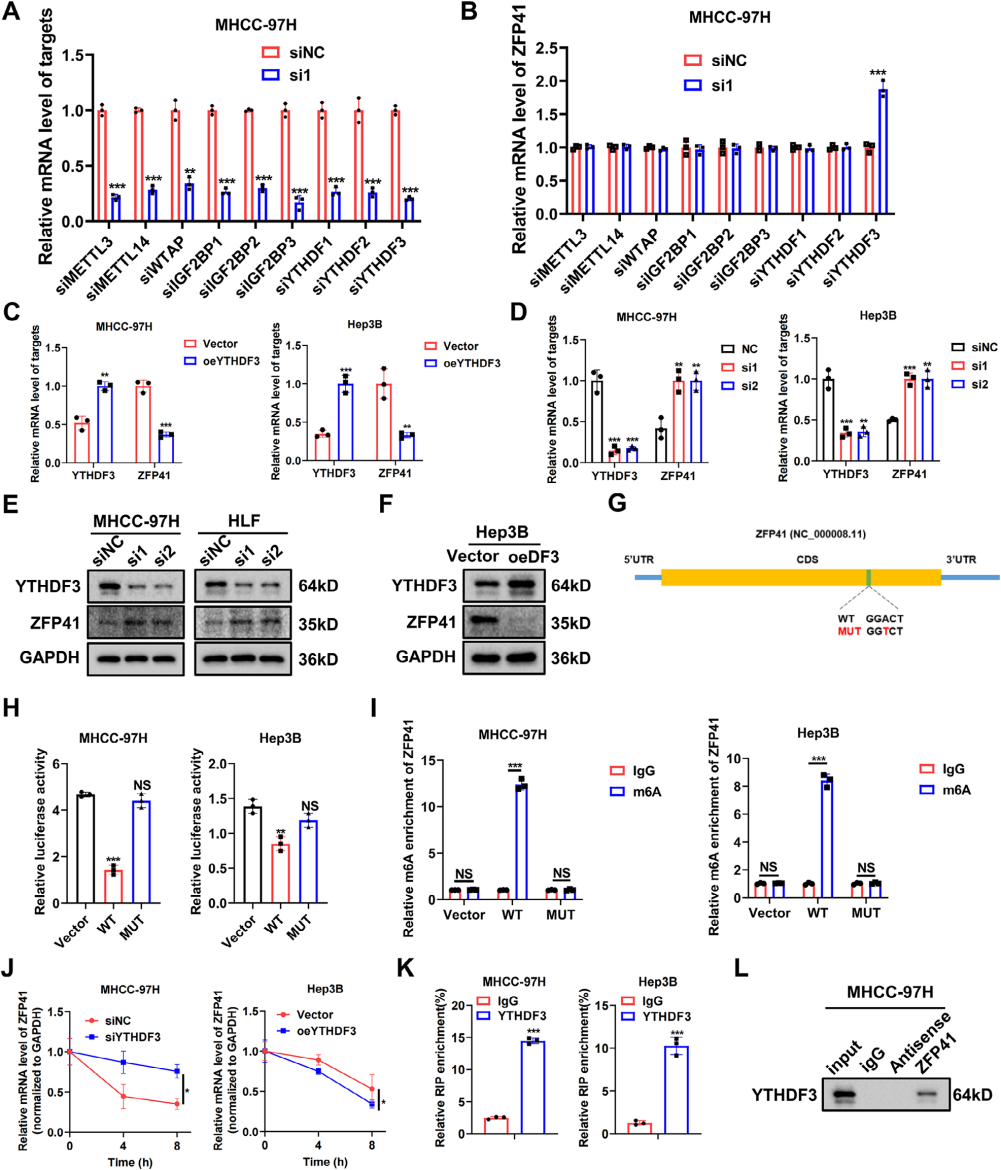
YTHDF3‐mediated m^6^A modification of ZFP41 mRNA and decays its mRNA stability. (A) qPCR results showed that efficiencies of siRNA‐mediated knockdown of common m^6^A regulators in MHCC‐97H cells. (B) qPCR results showed that expression of ZFP41 after silencing these m^6^A regulators in MHCC‐97H cells. (C) Overexpression of YTHDF3 notably suppress ZFP41 expression on transcription level in MHCC‐97H and Hep3B cells. (D) Knockdown of YTHDF3 obviously increase ZFP41 expression on mRNA level in MHCC‐97H and Hep3B cells. (E) Western blots results demonstrated that the ZFP41 protein level in MHCC‐97H and HLF cells after silencing YTHDF3 with siRNA of YTHDF3. (F) Western blots results demonstrated that the ZFP41 protein level in Hep3B cells after overexpression of YTHDF3. (G) The diagram of the potential site of m^6^A modification on the CDS (Coding Sequence) area. (H) The luciferase activity in both MHCC‐97H and Hep3B cells cotransfected with relative plasmids. (I) MeRIP results showed that ZFP41‐wt or ZFP41‐mut in both MHCC‐97H and Hep3B cells. (J) The rate of ZFP41 mRNA degradation in MHCC‐97H and Hep3B cells with YTHDF3 overexpression or knockdown. (K) The binding of ZFP41 mRNA and YTHDF3 was tested in MHCC‐97H and Hep3B cells by RIP‐qPCR analyses. (L) The relationship between ZFP41 and YTHDF3 was confirmed by RNA pull‐down assay.

As the interest in RNA modifications continues to grow, more research reporting on the molecular mechanism of m^6^A in HCC has arised.^17^ The m^6^A modification is initiated or removed by “writers” or “erasers” respectively, while “readers” specially bind m^6^A‐modified RNA and regulate RNA stability.^18^ To determine which the m^6^A regulator might be involved in regulation of the ZFP41 expression, we analyzed the mRNA level of ZFP41 in MHCC‐97H and HLF cells after siRNA‐mediated knockdown of several common m^6^A regulators, including METTL3, METTL14, WTAP, IGF2BPs, and YTHDFs. The result suggested that only si‐YTHDF3 caused a rise to an in ZFP41 mRNA level, while the others showed no significant effects (Figures 4A,B and S4A,B). We then assessed the mRNA level of ZFP41 after transfecting siRNA targeting YTHDF3 and overexpressing YTHDF3 in MHCC‐97H and Hep3B cells. RT‐qPCR results indicated that ZFP41 mRNA level was significantly upregulated upon YTHDF3 inhibition, whereas overexpression of YTHDF3 resulted in downregulation of the ZFP41 mRNA level (Figure 4C,D). Western blot analysis demonstrated that reducing YTHDF3 level led to an elevation in ZFP41 protein in MHCC‐97H and HLF cells, whereas increasing YTHDF3 expression led to a reduction in ZFP41 protein in Hep3B cells (Figure 4E,F).

On the basis of on‐line prediction of SRAMP (http://www.cuilab.cn/sramp) on ZFP41 mRNA, a putative m^6^A site was showed with high confidence (Figure 4G). To validate this predicted site, a point mutation was introduced into the site to further construct a luciferase reporter plasmid containing the wt or the mutants of ZFP41. The results indicated that YTHDF3 failed to change the luciferase activity when the mutant was present (Figure 4H). Moreover, the m^6^A‐specific antibody was found to be significantly enriched in ZFP41‐wt but was not mutant in MHCC‐97H and Hep3B cells (Figure 4I). In addition, the RNA decay assay validated that the stability of ZFP41 mRNA increased upon knockdown of YTHDF3 in MHCC‐97H cells, while it decreased following the overexpression of YTHDF3 in Hep3B cells (Figure 4J). Moreover, through RIP‐qPCR results, the binding of ZFP41 RNA to YTHDF3 in MHCC‐97H and Hep3B cells was identified (Figure 4K). Then, the RNA‐pull down assay was conducted to detect the physical binding of ZFP41 and YTHDF3 in these two cell lines, which was confirmed by the results of western blot assays (Figures 4L and S4C). Collectively, these findings revealed that YTHDF3 could directly bind to ZFP41, supporting the inhibition role of YTHDF3 in the regulation of ZFP41.

### 2.5 Snail is transcriptionally repressed by ZFP41 in HCC cells

**FIGURE 5:**
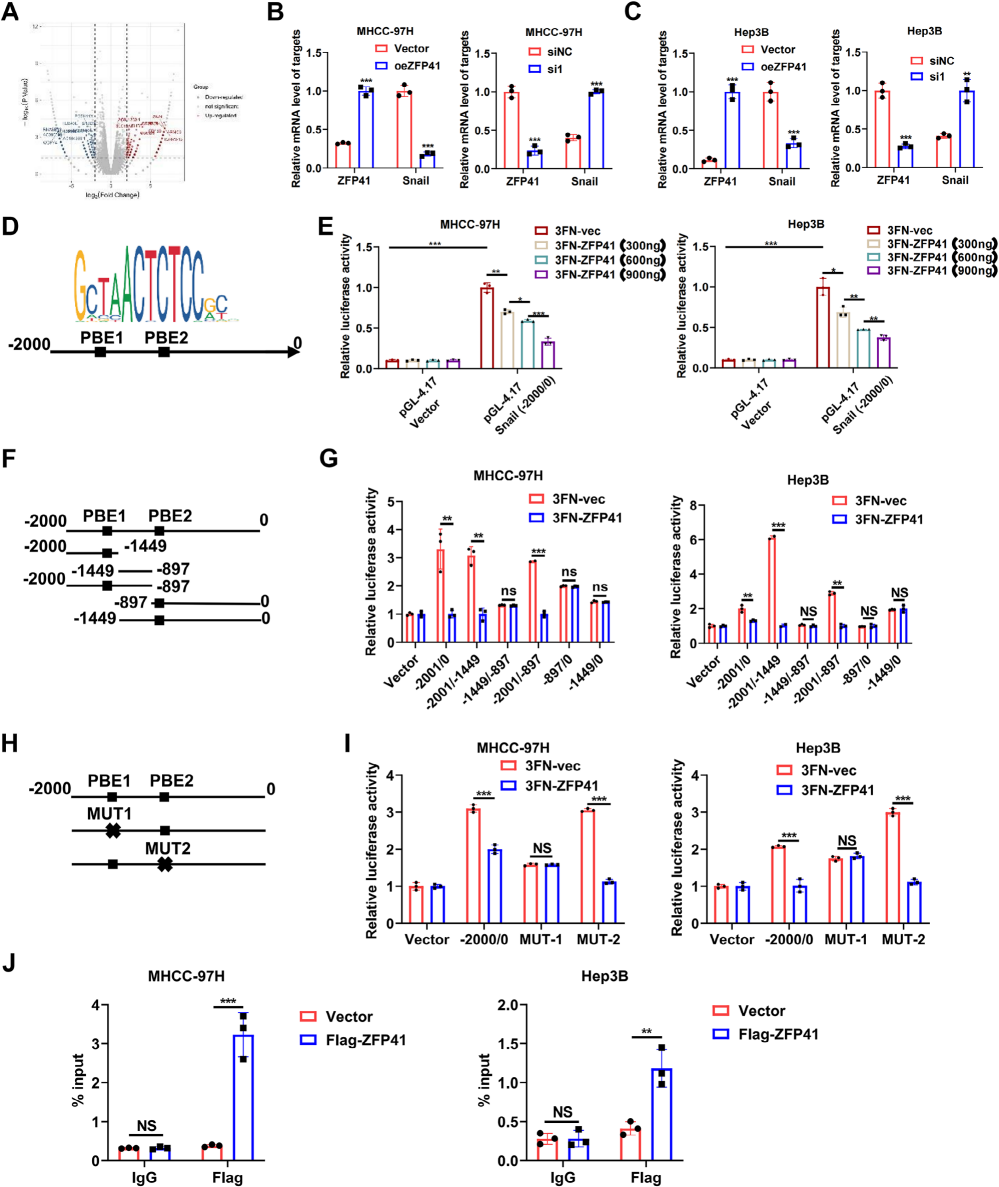
Snail is transcriptionally repressed by ZFP41 in HCC cells. (A) The volcano plot of differentially expressed genes in RNA‐sequencing. (B and C) qPCR analysis of Snail expression when overexpression or silencing ZFP41 in MHCC‐97H and Hep3B cells. (D) The JASPAR database demonstrated the potential binding sites of ZFP41 in the promoter of Snail. (E) MHCC‐97H and Hep3B cells were transfected with pcDNA3.1‐vec or ZFP41 at different dose, and relative luciferase activities of pGL4.17‐vec and wild type Snail promoter (−2000/0) were measured. (F) The diagram of truncated area on promoter of Snail. (G) Luciferase activities of wild type Snail promoter and truncated mutation in MHCC‐97H and Hep3B cells were measured and analyzed. (H) The diagram of mutated area on promoter of Snail. (I) Luciferase activities of wild type Snail promoter and mutations of the two possible binding elements in MHCC‐97H and Hep3B cells were measured and analyzed. (J) Anti‐FLAG‐ZFP41 chromatin immunoprecipitation assays were performed to validate the interaction between ZFP41 and Snail promoter.

The ZFP family is one of the largest transcription factor families.^19^ To explore how ZFP41 affects HCC progression, RNA‐sequencing (RNA‐seq) was conducted on MHCC‐97H cells with ZFP41 knockdown (Figure 5A). We identified and verified some differentially expressed genes through qPCR assays (Figure S5A). Notably, Snail, a key player in the EMT pathway across multiple tumors, appeared in our results. To confirm whether ZFP41 could regulate Snail, the qPCR assays were performed in MHCC‐97H and Hep3B cells overexpressing ZFP41. The mRNA level of Snail was decreased upon ZFP41 overexpression, while knocking down ZFP41 led to an upregulation of the Snail mRNA level in both two cell lines (Figure 5B,C). The JASPAR 2022 (https://jaspar.genereg.net/) prediction further showed the presence of two potential binding elements for ZFP41 within the −2000 to 0 bp region of the Snail promoter (Figure 5D). To validate the binding sites of ZFP41 on the Snail promoter, a luciferase reporter plasmid (PGL4.17) was constructed by cloning the promoter region of Snail from nucleotide −2000 to 0 bp. Luciferase reporter assays revealed that the upregulation of ZFP41 boosted the luciferase activity of Snail promoter in a dose‐dependent manner (Figure 5E). Five different truncated PGL4.17 plasmids were created and cotransfected into MHCC‐97H and Hep3B cells with ZFP41 and negative control to confirm the precise regions of ZFP41 interaction the Snail promoter. The results revealed that ZFP41 bound to the regions from −2001 to ‐897 bp (−2001/−897) and −2001 to ‐1449 bp (−2001/−1449) (Figure 5F,G). Therefore, it was hypothesized that ZFP41 might have a binding site at PBE1 in the Snail promoter. To test this hypothesis, we generated a mutation into the two different potential binding sites in the promoter, respectively. The results demonstrated that mutation of PBE1 eliminated the decrease of luciferase activity; however, mutation of PBE2 resulted in no difference compared with the complete promoter. These data suggested that ZFP41 could bind to the promoter of Snail at PBE1 (Figure 5H,I). Subsequently, CHIP assay was performed to investigate whether ZFP41 directly binds to the promoter of Snail in MHCC‐97H and Hep3B cells (Figure 5J). Overall, these results suggested that ZFP41 transcriptionally repressed the Snail expression in HCC cells.

### 2.6 ZFP41 suppresses the proliferation and invasion of HCC cells by inhibiting the Snail expression and EMT pathway

**FIGURE 6:**
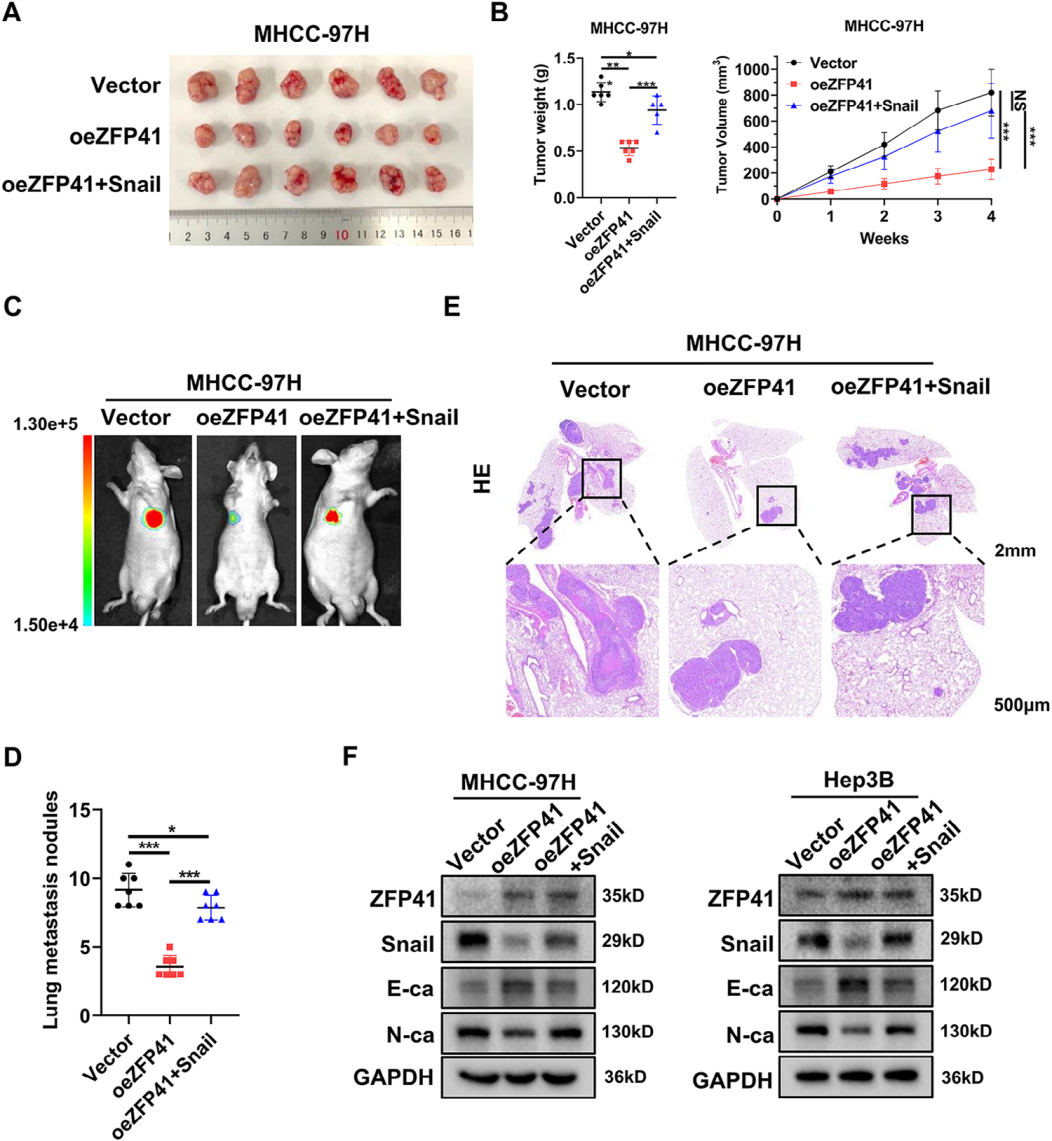
ZFP41 suppresses the proliferation and invasion of HCC cells by inhibiting Snail expression and EMT pathway. (A) Representative images of subcutaneous xenograft tumors formation of the Vector group, oeZFP41 group and oeZFP41+Snail group in MHCC‐97H cells. The dissected tumors from two groups were photographed. (B) Volumes and Weights of subcutaneous xenograft tumors in the Vector group, oeZFP41 group and oeZFP41+Snail group. (C and D) The lung fluorescence and the number of lung metastatic nodules was calculated in each group. (E) HE staining of lung tissue in each group were presented. (F) Western blots results showed the protein level of Snail and EMT‐related targets with Vector, oeZFP41, and oeZFP41+Snail groups in MHCC‐97H and Hep3B cells.

To examine whether ZFP41 regulates the repression effects by inhibiting the Snail expression in HCC cells, we detected the protein levels of Snail and EMT pathway‐related targets in Hep3B cells overexpressing ZFP41 and MHCC‐97H cells with ZFP41 knockdown were detected. The findings indicated that ZFP41 overexpression elevated E‐cadherin protein level and reduced Snail and N‐cadherin levels. Knockdown of ZFP41 resulted in the opposite results (Figure S5B). To test whether ZFP41 functions as a cancer suppressor through Snail, we established cell lines in which MHCC‐97H stably expresses ZFP41, as well as stably expressing both ZFP41 and Snail. First, we started with some in vitro assays. We transfected pcDNA3.1‐vector and ZFP41 into MHCC‐97H and Hep3B cells, or cotransfected pcDNA3.1‐ZFP41 and Snail into the same cells. The result indicated that increased Snail expression restored cell migration and invasion capabilities in both MHCC‐97H and Hep3B cells (Figure S6A). Similarly, the wound healing assay revealed the similar effects (Figure S6B). CCK8 assays and colony formation assays demonstrated that overexpression of ZFP41 suppressed the HCC cells proliferation ability, while overexpression of Snail abolished the suppressive effects of ZFP41 overexpression (Figure S6C–F). Next, we constructed different xenograft models. First, MHCC‐97H cells with overexpressing Vector, ZFP41 and both ZFP41 and Snail were subcutaneously injected in nude mice. After 4 weeks, the group with ZFP41 overexpression showed smaller tumor volume and tumor weight. Rescued tumor volume and tumor weight were showed in the overexpressing ZFP41+Snail group (Figure 6A,B). To evaluate the impact of ZFP41 on HCC metastasis in vivo, we injected MHCC‐97H cells with overexpressing Vector, ZFP41 and both ZFP41 and Snail through the tail vein in nude mice. After 6 weeks, the mice injected with ZFP41 overexpressed cells showed less lesions compared with those injected with control cells, and the overexpressing ZFP41+Snail group showed the rescued results compared with the overexpressing ZFP41 group (Figure 6C,E); the number of nodules in groups showed the same trend (Figure 6D). Furthermore, western blot assay results suggested that overexpression of ZFP41 increased the protein level of E‐cadherin, but decreased Snail and N‐cadherin protein levels. After overexpressing Snail, the relative protein levels were restored (Figure 6F). Consistent trends were also observed in these targets through IHC staining (Figure S2C,D).

Overall, above findings demonstrated that ZFP41 inhibits the EMT pathway by transcriptionally suppressing Snail, which in turn inhibits the proliferation and metastasis of HCC cells.

## 3 DISCUSSION

HCC remains one of the deadliest forms of cancer worldwide, and primary liver cancer accounts for the majority of liver cancers. Despite the diverse treatment options available for liver cancer, effective treatments beyond surgical resection are unavailable. Dysregulation of ZFPs is closely associated with the mechanisms involved in HCC progression.^20^, ^21^, ^22^ For example, ZEB1 has been shown to promote the EMT progression via its ability to induce transcriptional regulation in cervical cancer.^23^ In our study, ZFP41 was found to inhibit the transcriptional activity of Snail by exerting its role as a transcriptional repressor, thereby inhibiting the progression of EMT.

The field of epigenetics has gained growing interest in cancer research, particularly focusing on RNA m^6^A modification, which is among the most common RNA alterations studied. This modification involves various players, including “writers,” “erasers,” and “readers,” each playing different roles in the process. Among these regulators, METTL3, a crucial “writer,” has been widely reported in various cancers. Conversely, ALKBH5, which functions in an opposite manner to METTL3, has also been consistently reported in recent years.^24^, ^25^, ^26^, ^27^, ^28^ To discover the specific m^6^A regulator that affects the ZFP41 mRNA level, we employed siRNAs targeting METTL3, METTL14, WTAP, IGF2BPs, and YTHDFs, which are known regulators of m^6^A modification. According to qPCR results, only downregulation of YTHDF3 led to an increase in ZFP41 mRNA level. This result was unexpected, in previous studies related to m^6^A, a “Writer” typically modifies the mRNA of the target gene, subsequently stabilizing or degrading the mRNA via the “Reader.” Based on repetitive experimental data, we hypothesize this might be due to an unrecognized m^6^A regulator that then results in YTHDF3 degrading the mRNA of ZFP41. The details of this process warrant further investigation in our future work. Previous research reported the function of YTHDF3 in promoting the development of HCC through its m^6^A modification ability.^29^ Combined with our aforementioned results, we concluded that the low expression of ZFP41 in HCC is likely due to its degradation being mediated by YTHDF3.

To conclude, our research demonstrates that ZFP41 expression is reduced in tumor tissues of HCC patients relative to normal tissues, suggesting its potential as an independent prognostic element for HCC patients. Additionally, the findings revealed that increased level of ZFP41 could suppress the proliferation and metastasis of HCC cells both in vitro and in vivo. Given the breadth of m^6^A studies across many types of tumors, we set out to determine which m^6^A regulators are involved.^30^, ^31^, ^32^, ^33^, ^34^ After knocking down various m^6^A regulators, YTHDF3 was found to decay the mRNA level of ZFP41 through RNA decay assay. We also identified the specific site of m^6^A modification on ZFP41 using mutants and validated it through dual luciferase reporter assay and MeRIP‐qPCR. However, the underlying mechanism by which ZFP41 inhibits HCC progression remains unknown. To address this, RNA‐seq was applied to MHCC‐97H cells with ZFP41 knockdown and Snail was found to be one of most differentially expressed genes. The dual luciferase reporter assay revealed that ZFP41 inhibited Snail's transcription activity of in a manner dependent on dosage. We then constructed truncated and point mutant plasmids, and verified that ZFP41 regulates the specific site of Snail by dual luciferase reporter assay. Additionally, Chip‐qPCR assay revealed that ZFP41 could bind to the DNA of Snail, leading to its transcriptional repression. To further elucidate the role of Snail in the context of ZFP41, we performed experiments where Snail was on overexpressed alongside ZFP41, as showed in Figure 6.

**FIGURE 7:**
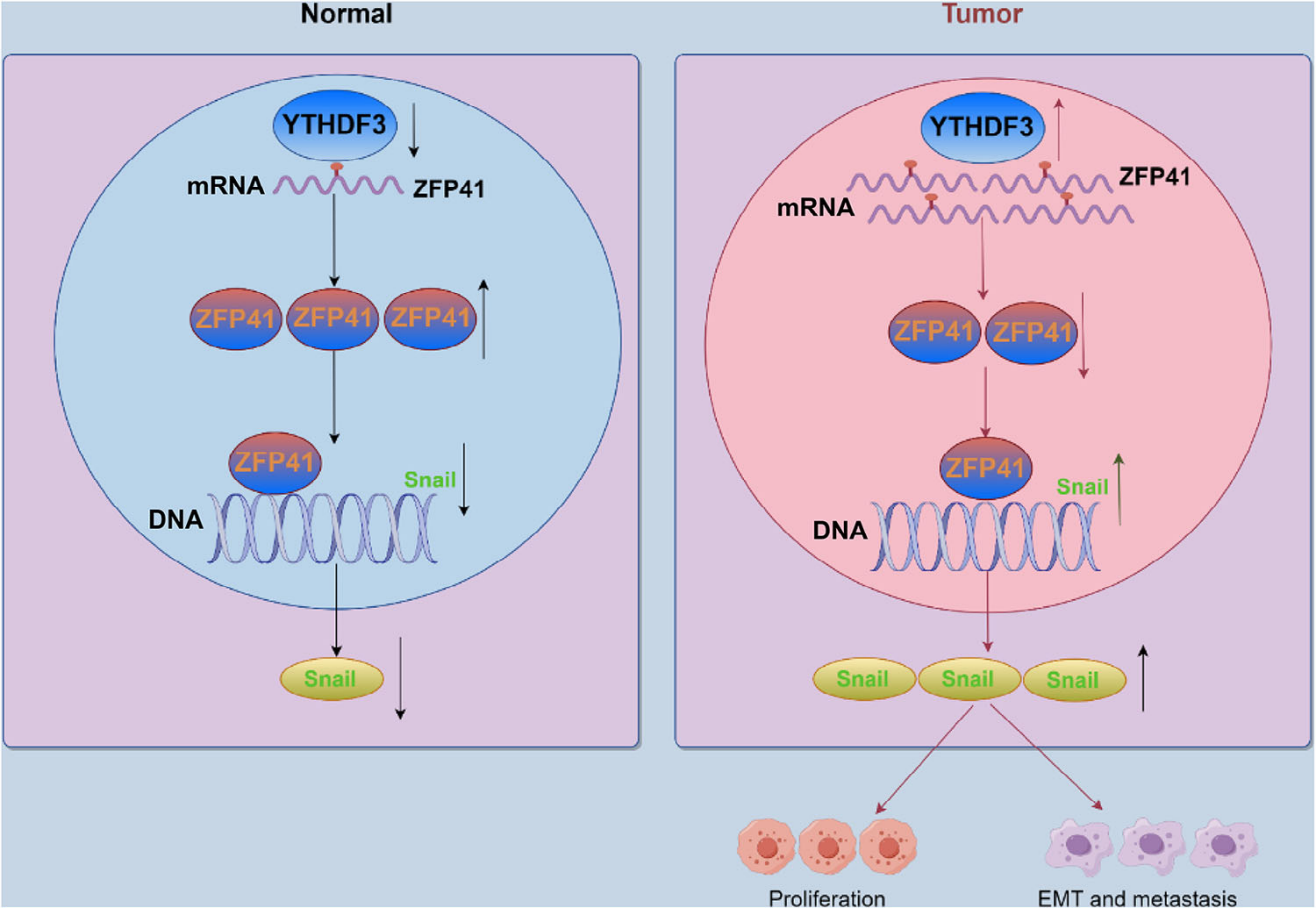
Schematic diagram of the mechanism showing the roles played by ZFP41 in HCC cells. In normal liver cells, YTHDF3 shows low expression, leading to a reduction in the m6A modification of ZFP41. This results in increased synthesis of ZFP41 mRNA and an enhanced transcriptional inhibitory effect on Snail. In contrast, in HCC cells, the expression of YTHDF3 is significantly elevated, which enhances the m6A modification of ZFP41. This promotes degradation of ZFP41 mRNA, consequently weakening the transcriptional inhibitory effect on Snail. Ultimately, this facilitates activation of the EMT pathway and promotes proliferation and metastasis of HCC.

Snail is a key molecular marker in the EMT pathway and is significantly elevated in numerous cancers, indicating a strong link with tumor metastasis.^35^, ^36^, ^37^, ^38^ In breast cancer, Snail has been shown to mediate cell migration by forming a complex with p66β to activate genes containing G‐box elements in promoters.^39^ Among the factors related to EMT, Snail acts as a key regulator of EMT‐associated genes like N‐cadherin, Vimentin, and E‐cadherin. Overexpression of Snail is commonly observed in HCC tissues. It contributes not only to cell migration and invasion but also confers stem cell‐associated characteristics to tumor cells and resistance to common treatments.^40^ In our studies, we have revealed that ZFP41 can bind to the DNA of Snail and inhibit its transcription activity. This discovery suggests that targeting the interaction between ZFP41 and Snail may offer a novel strategy for HCC therapy (Figure 7). By blocking or modulating this interaction, it may be possible to inhibit the pro‐metastatic functions of Snail and potentially overcome treatment resistance in HCC.

## 4 MATERIALS AND METHODS

### 4.1 Cell culture

All cell lines were kept as single‐layer cells in DMEM (Hyclone) supplemented with 10% fetal bovine serum (FBS) (Tsingmu, Wuhan, China) at 37°C in 5% CO_2_.

The anti‐sense oligonucleotides were designed and provided by AuGCT Biotecho to suppress the ZFP41 gene along with other m^6^A regulatory elements. Following the provided guidelines, siRNA was transfected using the Lipomaster 3000 Transfection Reagent (Vazyme, China). AuGCT Biotech (Wuhan, China) supplied the complete coding sequences of ZFP41, Snail, and YTHDF3.

### 4.2 Cell proliferation assay

The proliferation of HCC cells was assessed using the Cell Counting Kit 8. After cell transfection, the cells were evenly spread on 96‐well plates (1000 cells/well). Following the established procedure, cells were grown and the absorbance at 450 nm was measured at intervals of 24, 48, 72, 96, and 120 h.

### 4.3 Colony formation assay

MHCC‐97H, HLF, and Hep3B cells were seeded with six‐well plates (1500 cells/plate), and DMEM (10% FBS). Cells were cultured for 2 weeks, and the fluid was changed once every week. After a fortnight, the cells underwent three times washed with cold PBS, add 4% paraformaldehyde, and set for 15 min, then add crystal violet and stained for 15 min. The number of cells in each group was counted and analyzed.

### 4.4 Western blots and antibodies

The PVDF membrane (0.45 µm; Millipore) was blocked for 1 h at room temperature using TBST containing 5% BSA, then added the primary antibody, followed by incubation on a shaker at 4°C for 8 h. Then, the PVDF membrane was washed three times with TBST before being incubated for 1 h at room temperature with anti‐rabbit or anti‐mouse immunoglobulin G secondary antibody (Servicebio). Wash it three more times with TBST. Finally, the ECL development solution was used to identify the PVDF membrane. Antibodies for YTHDF3 (25537‐1‐AP), E‐cadherin (20874‐1‐AP), N‐cadherin (22018‐1‐AP), and GAPDH (60004‐1‐Ig) were supplied by Proteintech. Novus Biologicals provided the ZFP41 (NBP2‐83801) antibody. Cell Signaling Technology provided the Snail (#3879) antibody.

### 4.5 Immunohistochemistry

IHC staining was performed on the tissues with the kit provided by Servicebio. After dewaxing and rehydrating the paraffin sections, we performed antigen retrieval and blocked endogenous peroxidase. All steps are carried out in strict accordance with the instructions until the color detection is complete.

### 4.6 RNA stability assay

HCC cells were prepared by seeding them overnight in six‐well plates, followed by treatment with actinomycin D (5 µg/mL, HY‐17559; MedChemExpress) for durations of 0, 4, and 8 h. RNA extraction was performed with TRIzol after 36 h, and then qPCR was performed.

### 4.7 Dual‐luciferase report assays

The CDS length of ZFP41 gene, the mutant of the ZFP41 were cloned into the psiCHECK‐2 plasmid. Cells were plated at exceeding 60% density into 24‐well plates for 12 h, followed by cotransfected with the appropriate reagents. Thirty‐six hours after transfection, the relative activity was measured by the Dual Luciferase Reporter Assay Kit (DL101‐01; Vazyme).

### 4.8 Chromatin immunoprecipitation

ChIP experiment was performed using the CST Chromatin immunoprecipitation Kit. The cells were crosslinked with 1% formaldehyde at room temperature for 10 min, and then glycine was added to terminate the reaction. The cells are scraped off, centrifuged, collected, and washed. The collected cells were resuspended with Buffer A, then centrifuged to collect the nuclear precipitate, then resuspended with Buffer B for the first time, then centrifuged to collect the nucleus, and then resuspended with Buffer B for the second time. The second suspension with Buffer B requires a brief ultrasound treatment of the sample to allow complete chromatin release. FLAG antibody (F1804; Sigma–Aldrich) were then added to the sample and incubated at 4°C overnight, while IgG antibody (#2729; Cell Signaling Technology) was added to the control group as a negative control and incubated overnight at 4°C. Then, added Protein A/G magnetic beads and incubate together with the sample for 2 h at 4°C. After three rinses with low salt solution and one with high salt solution, the chromatin was eluted and cross‐linked, and the purified DNA was obtained by DNA purification column. Then, the qPCR assays were performed following the protocols. The specific CHIP primers are as follows: Snail‐F: TAAATTGACACGGGACGGGG, Snail‐R: CTGGTTCTAGCTGGAGAGCG.

### 4.9 Animal experiments

All animals are from GemPharmatech (Nanjing, China). All male BALB/c nude mice (4 weeks old) were allocated into Vec group, oeZFP41 group, shNC group, and shZFP41 group. The 1 × 10^6^ MHCC‐97H or HLF cells were injected under both arms to generate a subcutaneous xenograft model. After 3−4 weeks, the tumors were then weighed, photographed, and measured. In the lung metastasis model, luciferase bearing MHCC‐97H and Hep3B cells were injected into the tail vein of the BALB/C nude mice. Six weeks after the cells were injected, all the mice groups were sacrificed, and the intensity of luciferase bioluminescence were measured. Lung specimens were taken for HE section for subsequent data statistics

### 4.10 Statistical analysis

Each value was shown as the average plus or minus the standard deviation. Each experiment was performed three times. To assess statistical significance between two groups, Student's *t*‐tests (for normal distribution) or Wilcoxon signed‐rank tests (for matched pairs) were utilized. For multiple groups, statistical analysis was performed using either one‐way ANOVA or two‐way ANOVA. The Kaplan–Meier method illustrated the survival curves, while the log‐rank test evaluated their statistical significance. Correlations were evaluated using a Pearson correlation test. Appropriate statistical tests were conducted for all figures, with *p* < 0.05 considered statistically significant: *p < 0.05; **p < 0.01; ***p < 0.001; ns: not significant. All statistical values were calculated using GraphPad Prism 8.0 software.

## AUTHOR CONTRIBUTIONS

B. Z., Z. L., and X. C. conceived this study. B. Z. and Z. L. provided financial and administrative support. X. L. and M. H. conducted most experiments. H. Z. and H. L. collected the public data and patients’ data of Tongji cohort. X. L. and Z. L. wrote the manuscript. All authors have read and approved the final manuscript.

## CONFLICT OF INTEREST STATEMENT

The authors declare that they have no conflict of interest.

## ETHICS STATEMENT

*Human specimens*: The ethics committee of Tongji Hospital, affiliated with Tongji Medical College at Huazhong University of Science and Technology (Wuhan, China), granted approval for the study, a written informed consent was obtained from all patients for the use of their tissue samples (TJ‐IRB20211214).

*Mice*: All animal experimental procedures were also approved by the Committee on the Ethics of Animal Experiments of Tongji Hospital (TJH‐202209017).

## CONSENT FOR PUBLICATION

Not applicable.

## Supporting information



Supporting Information

## Data Availability

All data generated or analyzed during this study are included in this published article (and its supplementary information files). The data that support the findings of this study are available from the corresponding authors upon reasonable request. The raw sequence data have been deposited in Genome Sequence Archive in National Genomics Data Center, Beijing Institute of Genomics (https://ngdc.cncb.ac.cn/gsa‐human) with Project Accession No. PRJCA028794.
