# Supplementary material for: N6‐methyladenosine reader YTHDF3‐mediated zinc finger protein 41 inhibits hepatocellular carcinoma progression by transcriptional repression of Snail
Source: MedComm (2020). 2024 Oct 28;5(11):e763. doi: 10.1002/mco2.763 (PMC11518695; doi:10.1002/mco2.763)
Supplement: Supplementary file 1 — Supporting Information [file MCO2-5-e763-s001.docx]

**
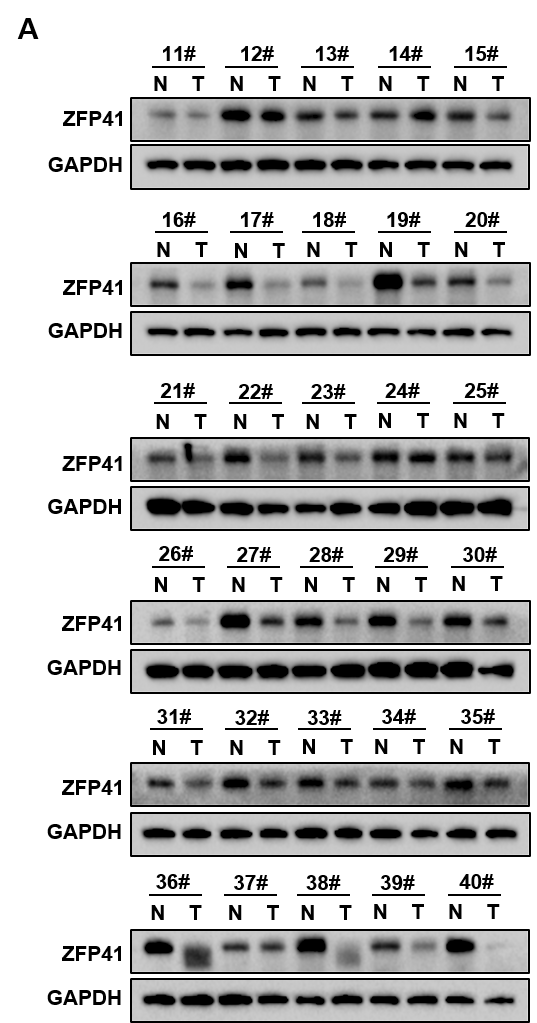
**

**Figure S1.** Downregulated ZFP41 is correlated with poor survival in HCC patients. (A) Western blots results demonstrated the 30 pairs of patient tissue samples.

**
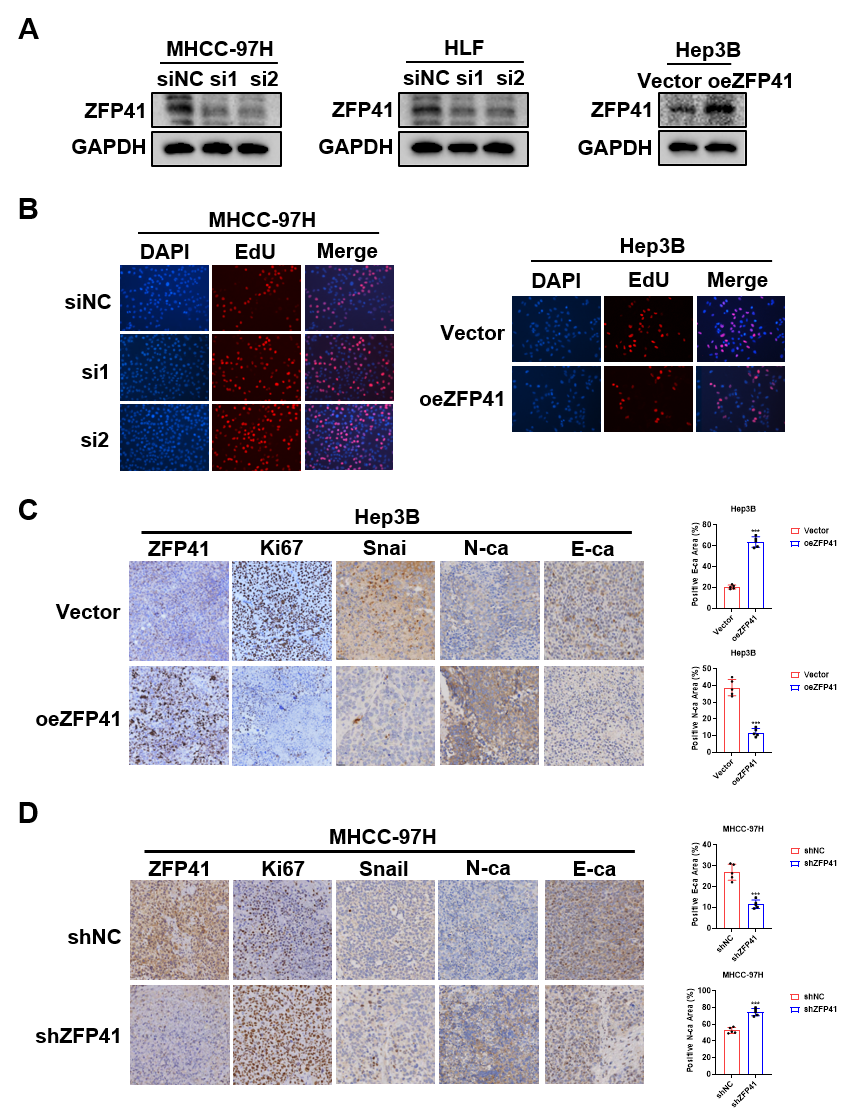
**

**Figure S2.** ZFP41 suppresses HCC cell proliferation in vitro and in vivo. (A) Western blots results showed knockdown and overexpression efficiency of ZFP41 protein. (B) The images of EdU in both MHCC-97H cells and Hep3B cells. (C-D) The IHC staining and relative statistical chart showed the protein expression of related targets in their groups.


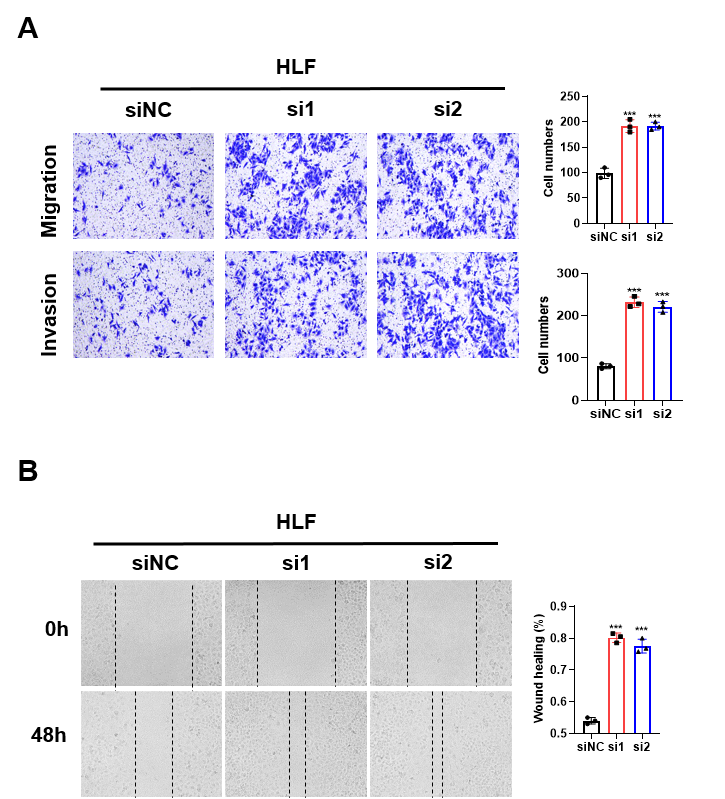


**Figure S3.** ZFP41 decreases HCC metastasis in vitro and in vivo. (A) The results revealed that knockdown of ZFP41 could promote cell migration and invasion in HLF cells. (B) Wound healing assays showed that similar effects.


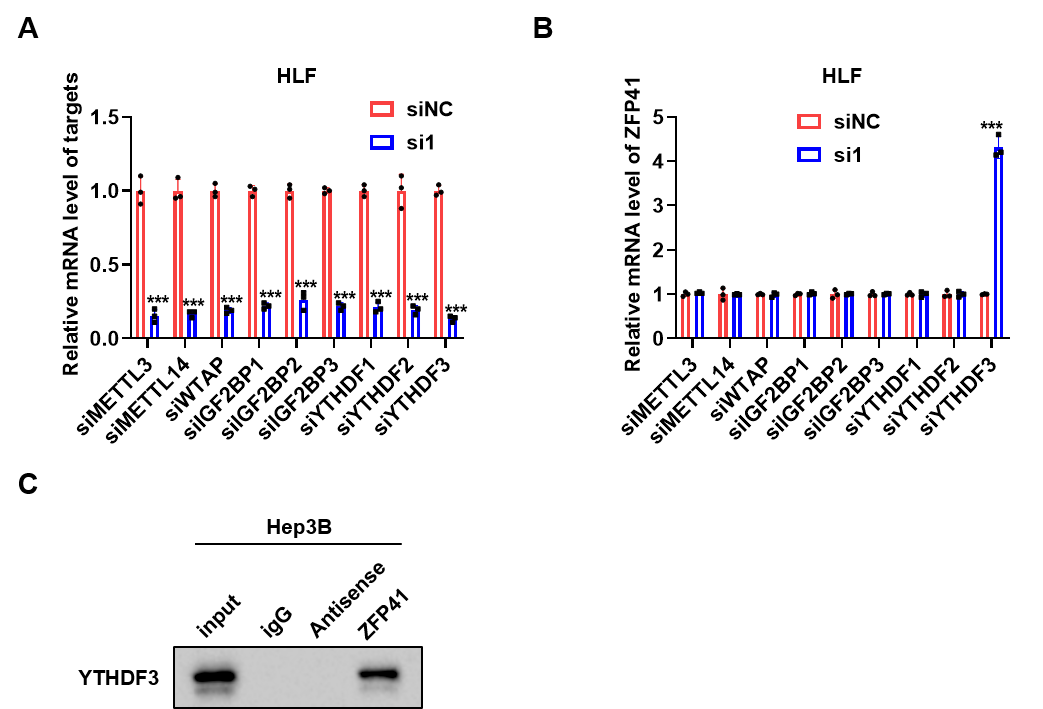


**Figure S4.** YTHDF3-mediated m6A modification of ZFP41 mRNA and decays its mRNA stability. (A-B) qPCR results showed that only knockdown of YTHDF3 could increase the mRNA level of ZFP41, the others showed no significances in HLF cells. (C) The RNA-pull down assay was performed to validate the physical connection between ZFP41 and YTHDF3 in Hep3B cells by western blotting.


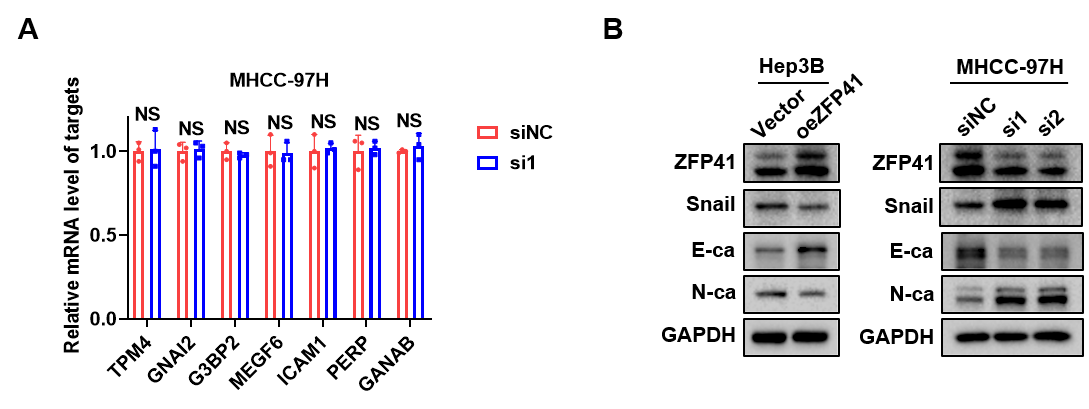


**Figure S5.** Snail is Transcriptionally Repressed by ZFP41 in HCC cells. (A) qPCR results showed that knockdown of ZFP41 did not change the mRNA level of targets in MHCC-97H cells. (B) Western blots results demonstrated that the protein level of Snail and the EMT-related targets after overexpression of ZFP41 or silencing ZFP41 in Hep3B and MHCC-97H cells.


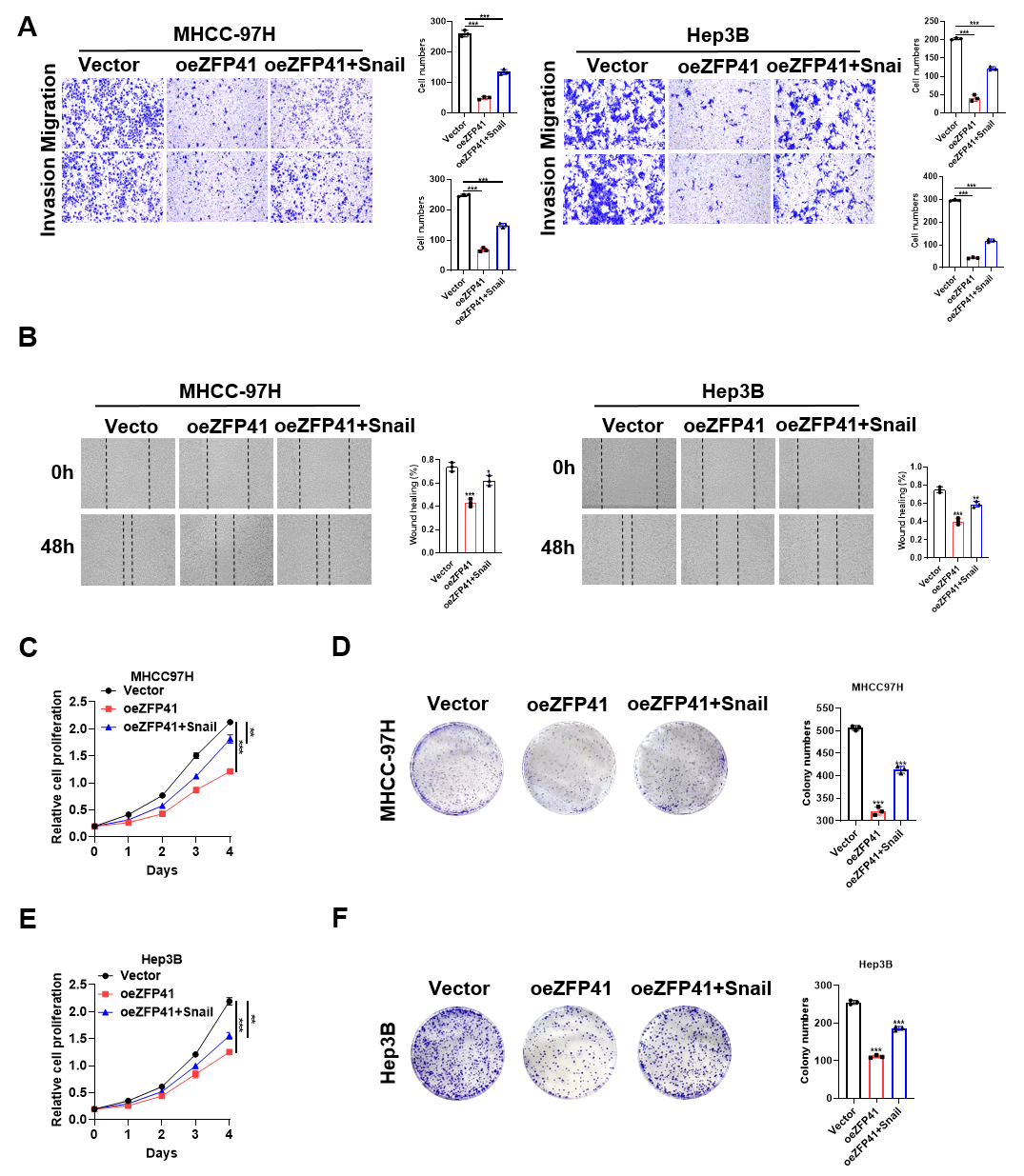


**Figure S6.** ZFP41 suppresses the proliferation and invasion of HCC cells by inhibiting Snail expression and EMT pathway. (A) The migration and invasion results with Vector, oeZFP41 and oeZFP41+Snail groups in MHCC-97H and Hep3B cells. (B) The wound healing assays with Vector, oeZFP41 and oeZFP41+Snail groups in MHCC-97H and Hep3B cells. (C, E) CCK-8 assays with Vector, oeZFP41 and oeZFP41+Snail groups in MHCC-97H and Hep3B cells. (D, F) Colony formation assays with Vector, oeZFP41 and oeZFP41+Snail groups in MHCC-97H and Hep3B cells. Data represent the MEAN±S.E.M. of three independent experiments. *P<0.05; **P<0.01; ***P<0.001; ****P<0.0001 (Student’s t test)

**
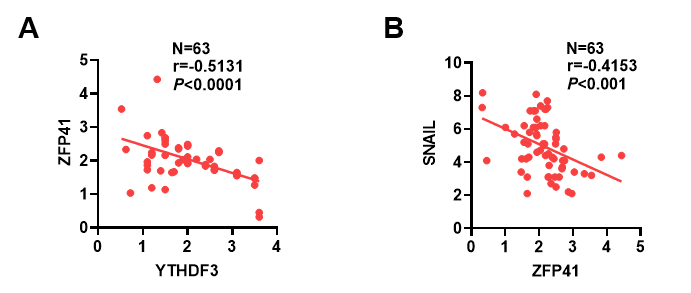
**

**Figure S7.** The correlation analyses between YTHDF3 and ZFP41, ZFP41 and Snail mRNA levels in HCC specimens. (A) The correlation of mRNA levels between YTHDF3 and ZFP41 in HCC specimens as shown in the figure. (B) The correlation of mRNA levels between ZFP41 and Snail in HCC specimens as shown in the figure.

**All original western blot images**

**
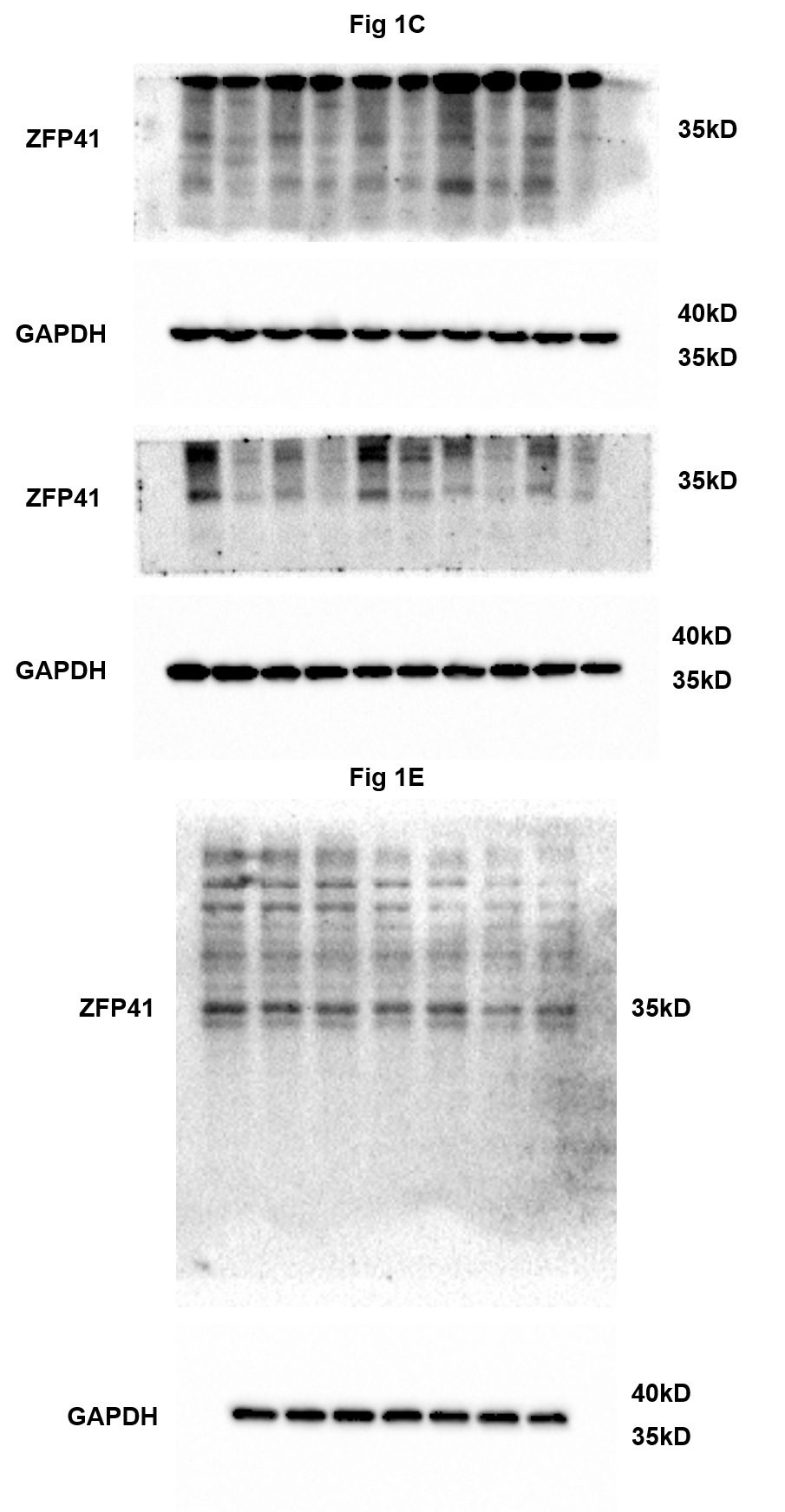

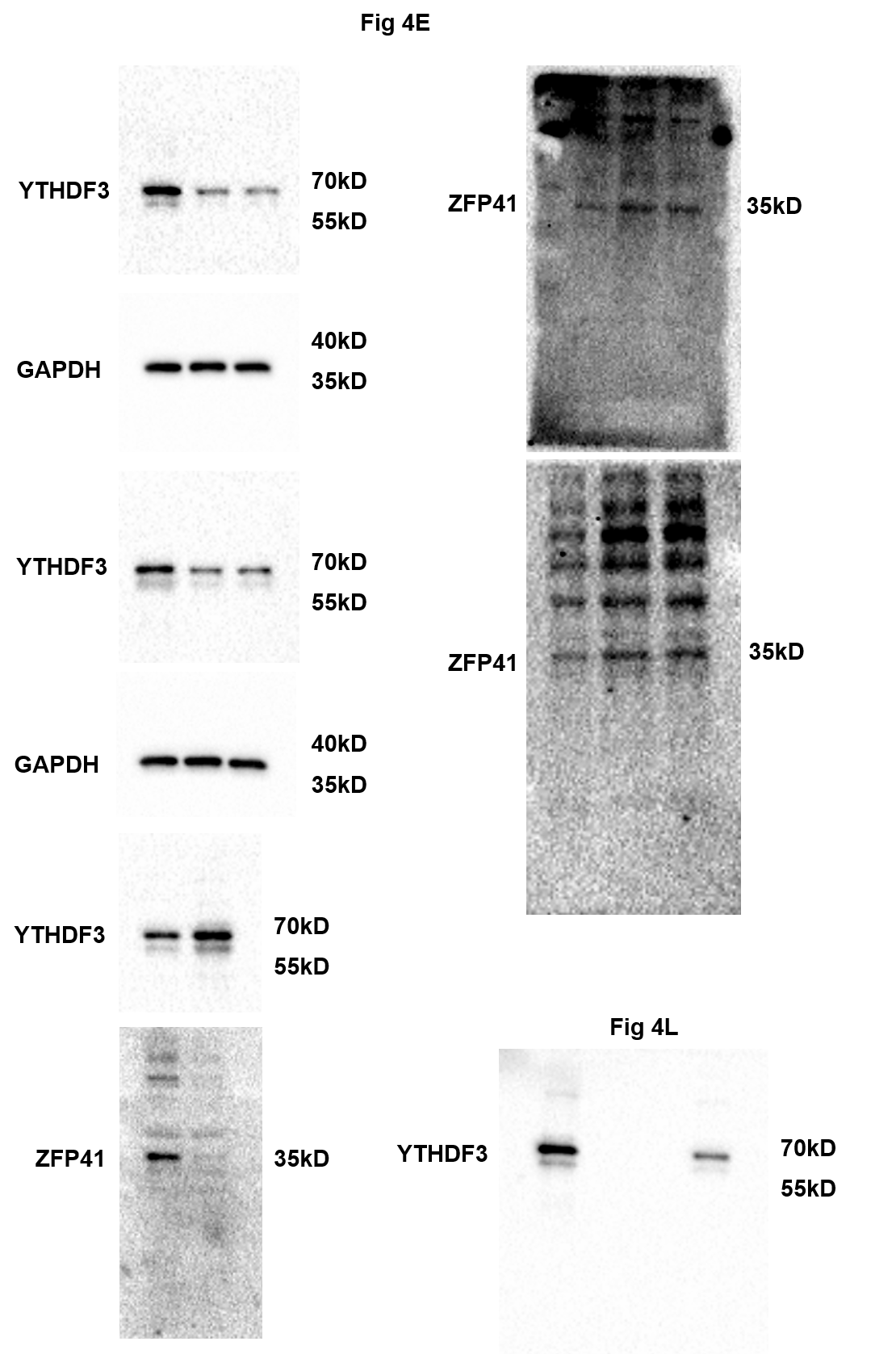
**

**
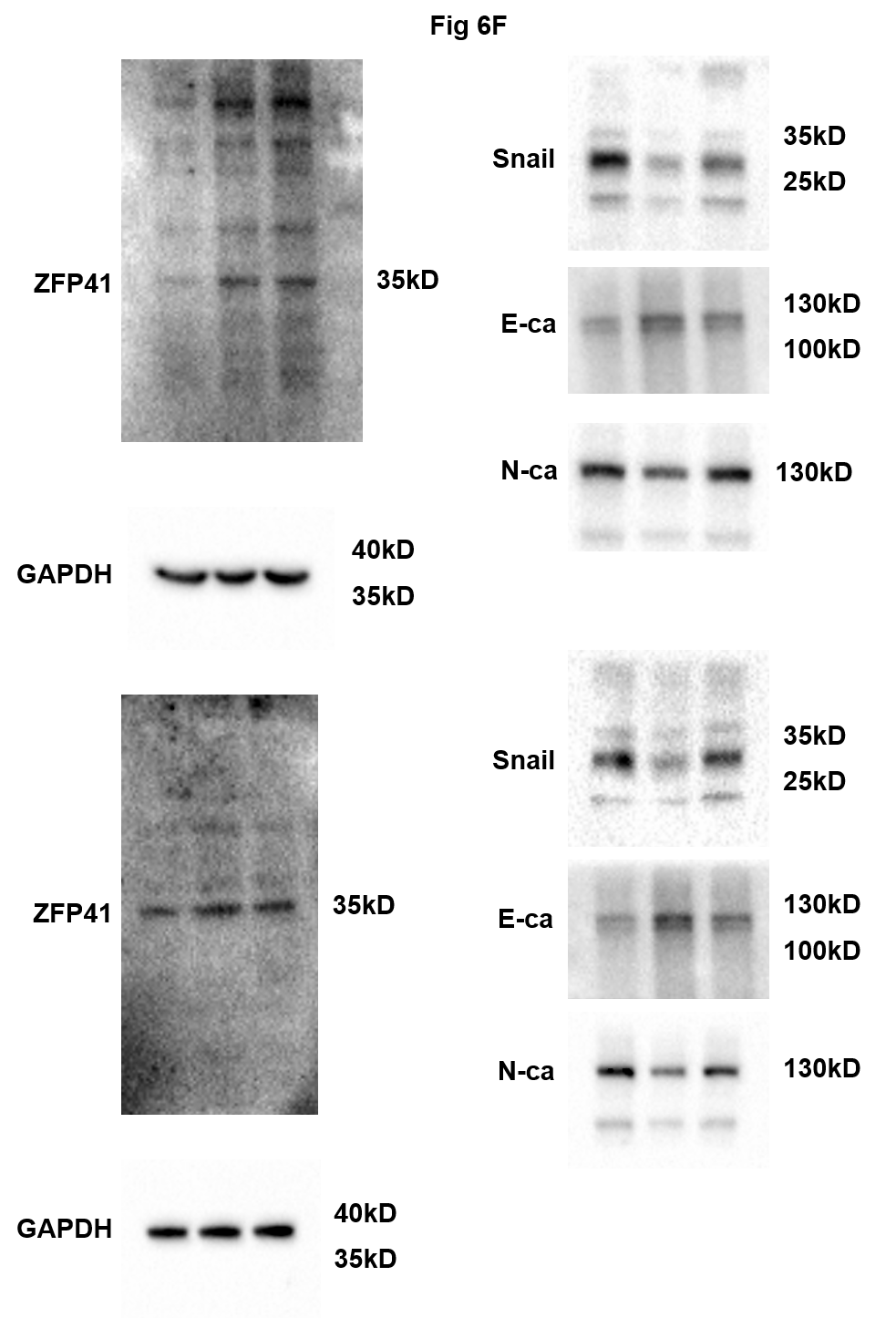
**

**
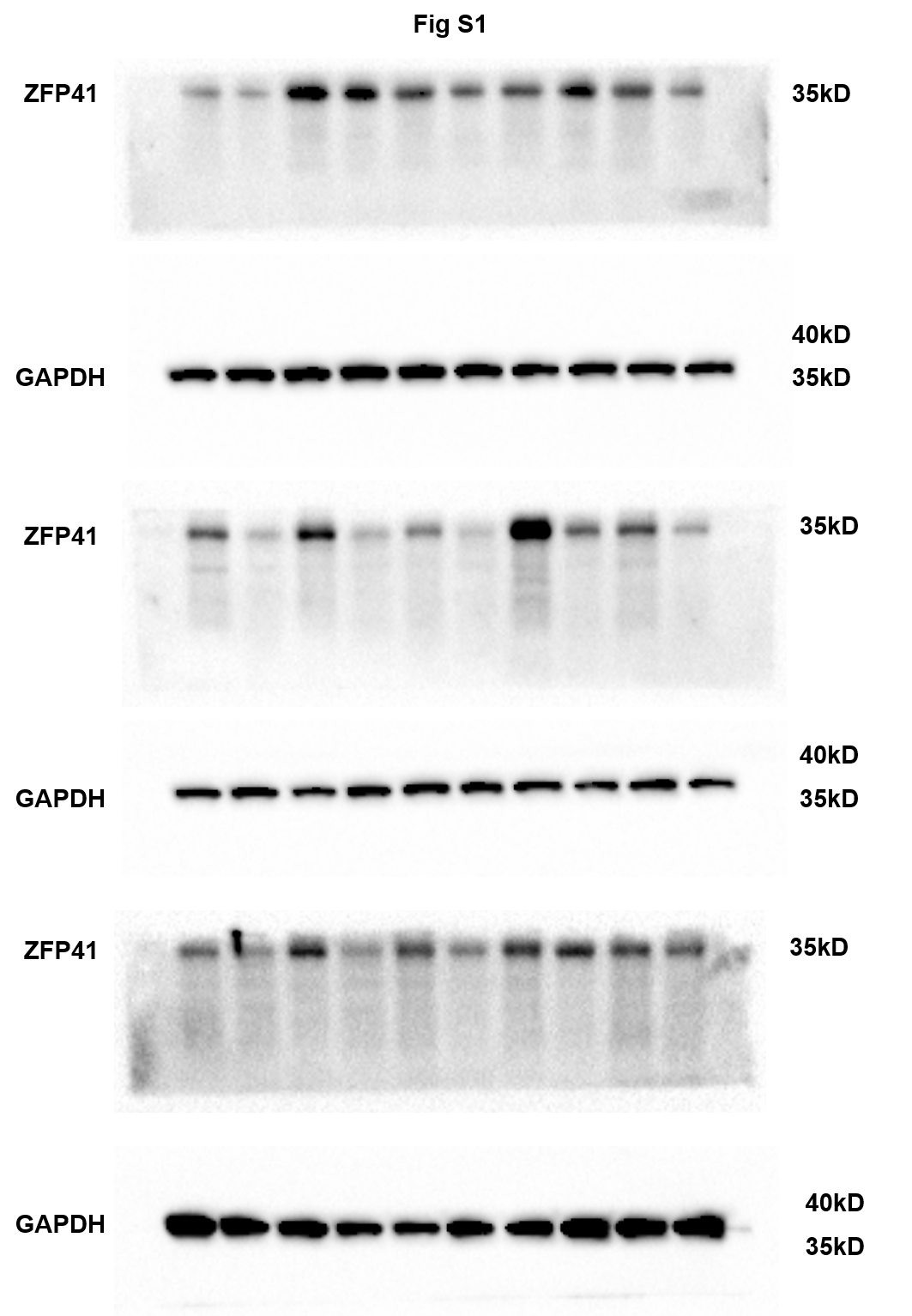
**

**
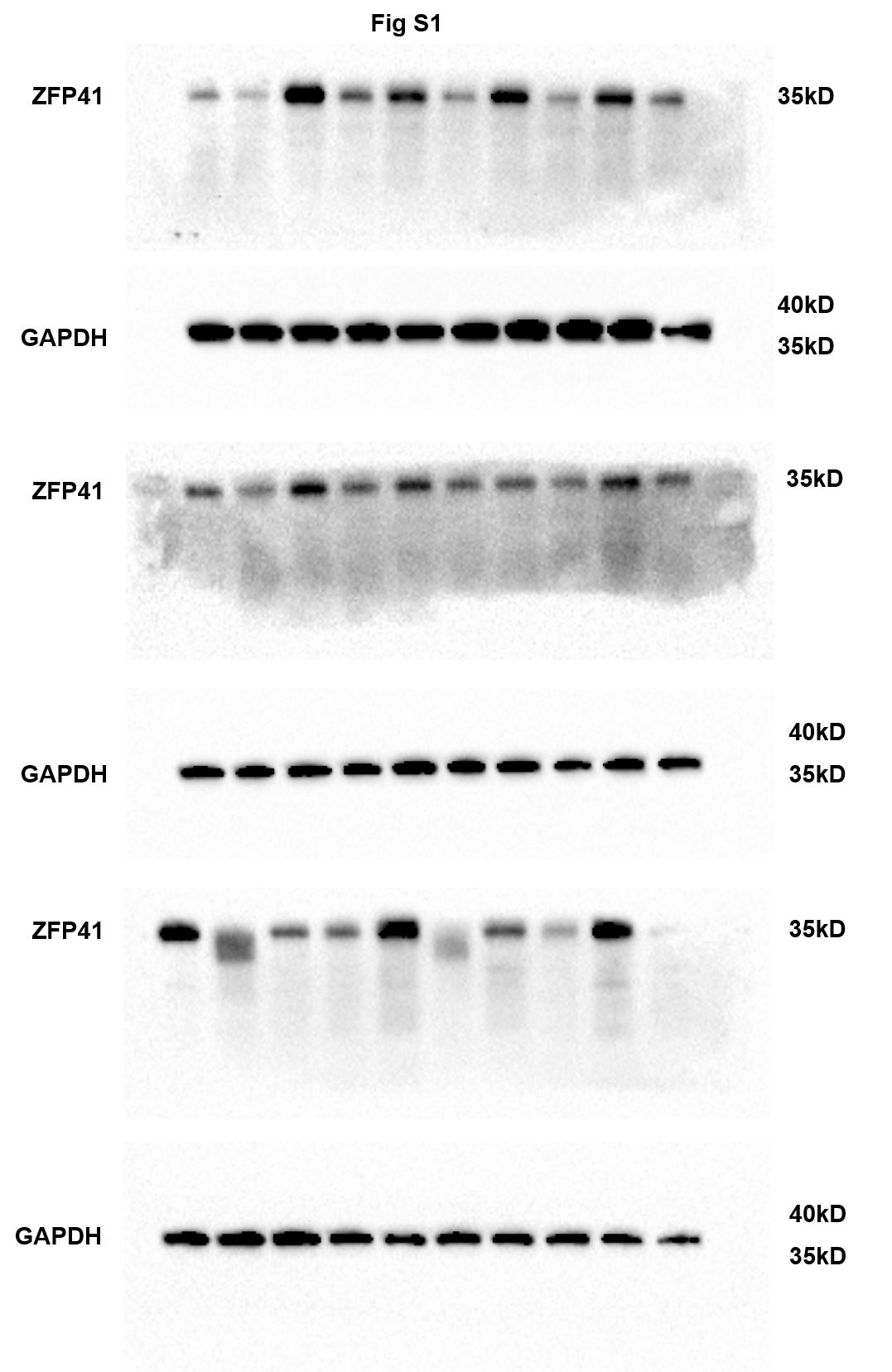
**

**
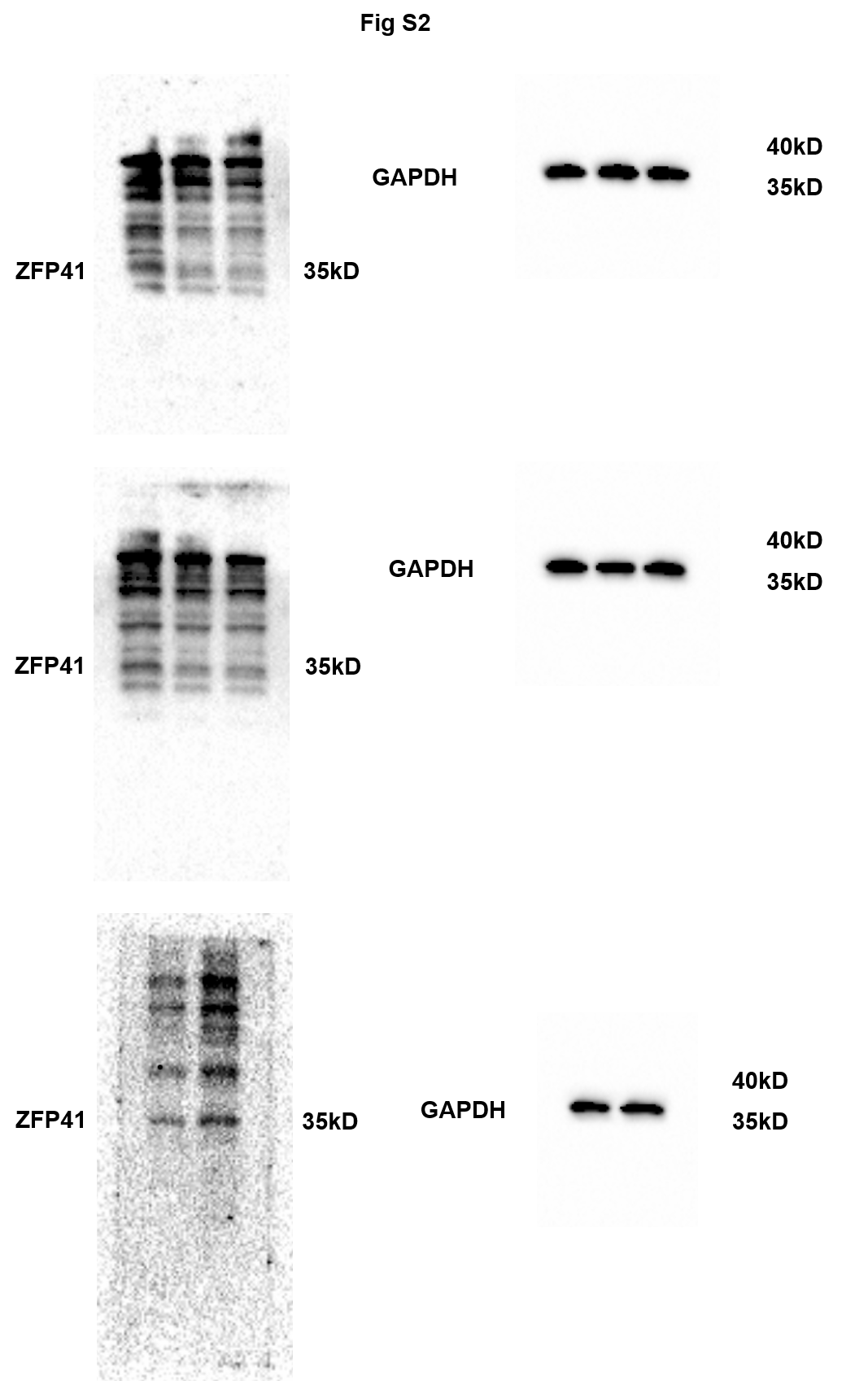
**

**
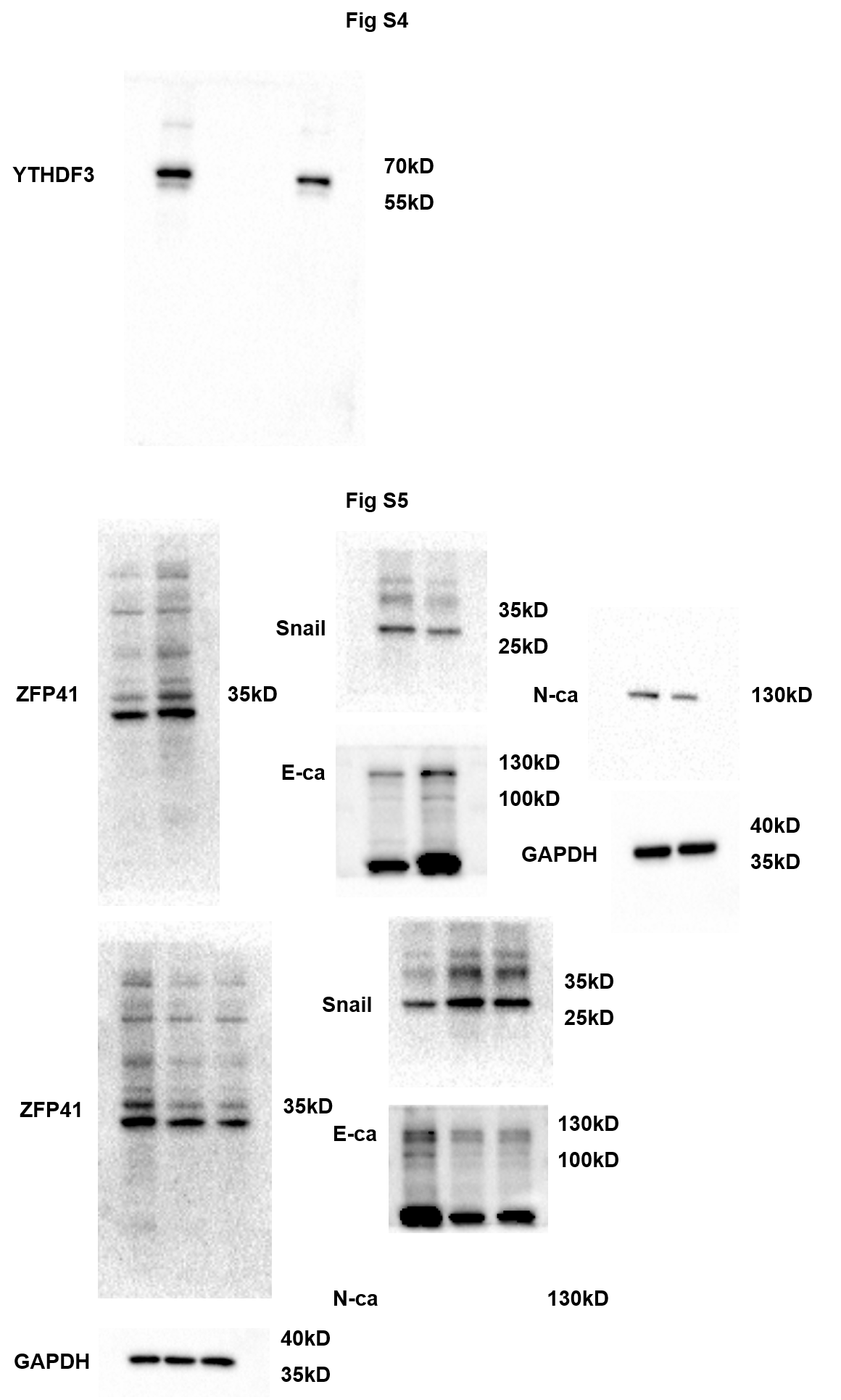
**

**Supplementary Table 1. Correlation between ZFP41 and clinicopathological characteristics in HCC (n=105).**

| **Clinicopathological**  **variables** | **Relative ZFP41 Expression** | | **P value** |
| --- | --- | --- | --- |
|  | **Low 42** | **High 63** |  |
| **Gender**  Male  Female | 36  6 | 50  13 | 0.407 |
| **Age**  ≤50  > 50 | 27  15 | 33  30 | 0.227 |
| **AFP (ug/L)**  ≤20  > 20 | 12  30 | 15  48 | 0.584 |
| **GGT(u/l)**  ≤54  > 54 | 23  19 | 25  38 | 0.128 |
| **ALT(ng/ml)**  ≤75  >75 | 12  30 | 27  36 | 0.137 |
| **Cirrhosis**  No  Yes | 24  18 | 23  40 | **0.037** |
| **Tumor size (cm)**  ≤5  >5 | 12  30 | 44  19 | 0.069 |
| **Tumor number**  Single  Multiple | 15  27 | 11  52 | **0.033** |
| **Vascular invasion**  No  Yes | 24  18 | 29  34 | 0.264 |
| **Differentiation**  I- II  III-IV | 15  27 | 29  34 | 0.293 |
| **BCLC stage**  0+A  B+C | 20  22 | 44  19 | **0.022** |

**Supplementary Table 2. The IHC scores of ZFP41 staining on tissue arrays that included 105 paired HCC specimens and normal tissues (n=105).**

| Normal | Tumor | Number of patient |
| --- | --- | --- |
| 4 | 2 | 942447 |
| 4 | 1 | 1342173 |
| 4 | 0 | 1005271508 |
| 4 | 1 | 1005257703 |
| 4 | 1 | 1005155781 |
| 4 | 2 | 1005126159 |
| 3 | 0 | 1005095847 |
| 4 | 1 | 1005043232 |
| 8 | 1 | 1005023935 |
| 4 | 0 | 1004937028 |
| 4 | 1 | 1004865672 |
| 4 | 1 | 1004849891 |
| 4 | 1 | 1004620117 |
| 4 | 3 | 1004445354 |
| 3 | 1 | 1004431029 |
| 5 | 1 | 1004427918 |
| 4 | 0 | 1004426366 |
| 4 | 2 | 1004416578 |
| 4 | 1 | 1004400886 |
| 4 | 1 | 1004391451 |
| 4 | 3 | 1004342300 |
| 4 | 2 | 1004308080 |
| 4 | 1 | 1004300087 |
| 8 | 1 | 1004290517 |
| 4 | 1 | 1004262738 |
| 3 | 2 | 1004229845 |
| 4 | 1 | 1004229333 |
| 4 | 2 | 1004228272 |
| 4 | 1 | 1004204835 |
| 3 | 0 | 1004075016 |
| 4 | 0 | 1003995265 |
| 4 | 3 | 1003850631 |
| 4 | 2 | 1003727630 |
| 4 | 1 | 1003581410 |
| 4 | 1 | 1003525299 |
| 4 | 1 | 1003495956 |
| 4 | 2 | 1003473141 |
| 5 | 0 | 1003443794 |
| 4 | 1 | 1003442573 |
| 4 | 1 | 1003158298 |
| 3 | 0 | 1002485242 |
| 2 | 0 | 1002418270 |
| 1 | 0 | 1001599100 |
| 4 | 1 | 1001418730 |
| 4 | 0 | 1005400271 |
| 4 | 1 | 1005394742 |
| 4 | 1 | 1005343538 |
| 6 | 4 | 1005338776 |
| 4 | 3 | 1005291014 |
| 4 | 2 | 1005269931 |
| 3 | 2 | 1005183592 |
| 6 | 2 | 1005182515 |
| 4 | 1 | 1005168251 |
| 4 | 0 | 1005160633 |
| 6 | 1 | 1005144883 |
| 4 | 1 | 1005105135 |
| 4 | 1 | 1005092815 |
| 4 | 0 | 1005075204 |
| 4 | 0 | 1004941112 |
| 4 | 1 | 1004804756 |
| 4 | 0 | 1004803302 |
| 5 | 2 | 1004794531 |
| 4 | 2 | 1004785863 |
| 4 | 0 | 1004728503 |
| 4 | 3 | 1004657466 |
| 4 | 0 | 1004656655 |
| 4 | 1 | 1004654149 |
| 4 | 3 | 1004578517 |
| 4 | 3 | 1004498230 |
| 4 | 1 | 1004495003 |
| 4 | 2 | 1004487659 |
| 4 | 1 | 1004447694 |
| 4 | 1 | 1004369924 |
| 4 | 1 | 1004331145 |
| 4 | 1 | 1004303482 |
| 4 | 1 | 1004229211 |
| 4 | 2 | 1003703656 |
| 4 | 0 | 1003508941 |
| 4 | 1 | 1003443624 |
| 4 | 0 | 1003408916 |
| 4 | 1 | 1003301294 |
| 4 | 1 | 1003300276 |
| 4 | 1 | 1003300046 |
| 4 | 2 | 1003059000 |
| 8 | 4 | 1002903958 |
| 4 | 0 | 1002228003 |
| 1 | 0 | 1001769916 |
| 4 | 1 | 1001769762 |
| 1 | 0 | 1001722920 |
| 3 | 0 | 1001694683 |
| 4 | 3 | 1001582060 |
| 4 | 0 | 1333440 |
| 1 | 0 | 1303277 |
| 4 | 1 | 908039 |
| 4 | 4 | 1005135178 |
| 4 | 4 | 1005061952 |
| 1 | 4 | 1004406723 |
| 4 | 4 | 1003566118 |
| 1 | 1 | 1002042122 |
| 4 | 5 | 1005004303 |
| 4 | 6 | 1004917836 |
| 4 | 4 | 1003811434 |
| 0 | 3 | 1003525660 |
| 2 | 2 | 1002366229 |
| 4 | 6 | 1001261250 |
